# Cellular interactions and Ion channel signatures in atrial fibrillation remodeling: insights from single-cell analysis and machine learning

**DOI:** 10.3389/fcvm.2025.1615574

**Published:** 2025-08-15

**Authors:** Bin He, Yan Cheng, Juan Wang, Ya Zhan, YanQun Liu

**Affiliations:** ^1^ The No. 1 Department of Gerontology, The Third Hospital of Mianyang, Sichuan Mental Health Center, Mianyang, China; ^2^ Renal Department, The Third Hospital of Mianyang, Sichuan Mental Health Center, Mianyang, China

**Keywords:** atrial fibrillation, structural remodeling, electrical remodeling, single-Cell analysis, machine learning

## Abstract

**Background:**

Atrial structural and electrical remodeling are the pathophysiological mechanisms underlying atrial fibrillation (AF). Although previous studies have offered insights into these changes, the cellular interactions involved in atrial structural remodeling and the ion channel marker genes associated with electrical remodeling in AF remain insufficiently elucidated.

**Methods:**

We used single-cell RNA sequencing (scRNA-seq) to investigate the structural remodeling in AF at the cellular level. Raw data from atrial fibroblasts of AF patients and controls were pre-processed using Seurat (R package), with stringent quality control to filter out low-quality cells. Differential gene expression and clustering were performed, followed by principal component analysis (PCA) to identify significant cell types. Cell trajectory analysis was carried out to explore the differentiation patterns of these fibroblasts using Monocle. Additionally, a cell-cell interaction analysis was performed using the CellChat package, and biological function and pathway enrichment analyses were done using GO, KEGG and GSEA pathways. Ion channel-related genes were extracted from microarray datasets and analyzed for differential expression and functional relevance to AF pathology. Machine learning algorithms (LASSO and SVM) were used to identify signature genes from ion channels in AF, followed by drug-enrichment analysis to explore potential therapeutic options.

**Results:**

In the structural remodeling investigation, single-cell analysis was employed to identify five distinct cell subtypes, including embryonic fibroblasts (EF), actively proliferating fibroblasts (APF), smooth muscle cells (SMC), endothelial cells (EC), and leukocytes (LBCs). These subtypes exhibited significantly different distributions between AF and SR. In the AF group, the proportions of EF, APF, and LBCs were increased, whereas the proportion of EC was decreased; by contrast, the SR group displayed a higher proportion of EC. Trajectory analysis suggested that EF cells in AF may differentiate into both APF and SMC subtypes. Cell–cell communication analysis revealed extensive signaling pathways (e.g., LAMININ and COLLAGEN) activated in EF cells under AF conditions, in addition to the specific activation of MK signaling in AF. It also uncovered a loss of certain EC signals (e.g., GRN–SORT1 and AGRN–DAG1) in AF and a marked reduction in NPPA–NPR1 signaling from SMC to EC. These findings indicate that such alterations may be crucial to the onset and maintenance of AF. In the electrical remodeling investigation, ion channel gene sets and gene expression data were utilized alongside LASSO and SVM machine-learning algorithms combined with ROC curve analysis. This approach ultimately identified *ANO1* and *GRIK2* as the characteristic ion channel genes for AF. Both genes demonstrated strong discriminative power in distinguishing AF from SR. Finally, drug-targeting analyses suggested that phenytoin sodium—a known antiarrhythmic agent—may exert therapeutic effects by targeting critical EF subtypes in AF. Moreover, ionomycin and DIDS were found to be strongly associated with *ANO1*, whereas *GRIK2* was linked to citalopram and topiramate.

**Conclusion:**

This study underscores the critical roles of cell distribution, cell developmental trajectories, and intercellular interactions in the structural remodeling of AF, as well as the key ion channel biomarkers involved in AF-related electrical remodeling. In terms of structural remodeling, the proportions of EF, APF, and LBCs are elevated in AF, with EF cells potentially differentiating into APF or SMC. Moreover, active EF cell signaling and the loss of EC signals in AF may be crucial for the onset and maintenance of this arrhythmia. Regarding electrical remodeling, *ANO1* and *GRIK2* have been identified as potential biomarker genes for AF. Notably, phenytoin sodium may exert therapeutic effects against AF by targeting EF subtypes. In addition, ionomycin, citalopram, and topiramate exhibit modulatory effects on ion channels, providing new potential treatment avenues. Such drug repurposing represents a rapid and efficient strategy for the discovery of novel AF therapies.

## Graphical Abstract

**Figure:**
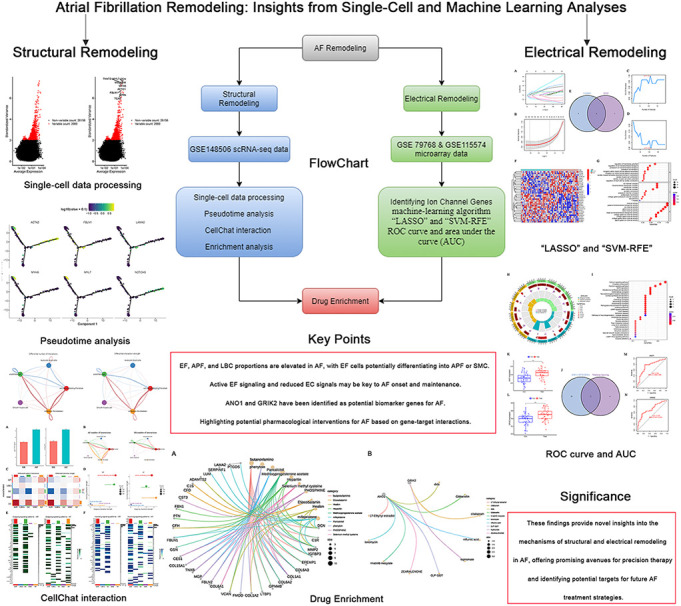

## Introduction

Atrial fibrillation (AF) is the most common arrhythmia, affecting an estimated 33.5 million patients worldwide. It is characterized by rapid and irregular atrial activation that impairs atrial function and predisposes individuals to a range of serious complications, including stroke, thromboembolism, heart failure, and cognitive dysfunction (1). These complications impose a substantial burden on both society and families. Reported risk factors for AF include age, sex differences, genetics, hypertension, cardiovascular diseases, and environmental factors. The diversity of these risk factors and the severity of their associated complications underscore the importance of developing effective interventions. However, current treatment approaches still face significant challenges. Although effective anticoagulants and left atrial appendage occlusion can reduce the risks of stroke and thromboembolism, and antiarrhythmic drugs and radiofrequency ablation can control rhythm and alleviate symptoms, drugs are often accompanied by notable side effects, while surgical interventions are invasive and carry serious complications. Therefore, developing novel therapeutic interventions is crucial for overcoming these challenges.

AF is a progressive disease often evolving from paroxysmal to persistent AF, with atrial remodeling playing a critical role in this progression. Atrial remodeling represents the common outcome of various risk factors and etiologies in AF, and serves as the primary pathophysiological mechanism. It mainly involves electrical remodeling and structural remodeling. In the early stages, electrical remodeling is marked by changes in electrophysiology and ion channel characteristics, whereas in later stages, structural remodeling is manifested by fibrosis of the atrial myocardium and extracellular matrix, as well as myocyte apoptosis and other tissue-structure alterations (2, 3). Among these, atrial electrical remodeling predominantly involves abnormalities in transmembrane ion channels, which are formed by specialized proteins (channels and transporters) that tightly regulate ion movement across cardiac cell membranes. Such ion channel protein dysfunction is closely linked to arrhythmogenesis, including L-type calcium channels, fast sodium channels, and various potassium channels, resulting in a shortened atrial effective refractory period and action potential duration. This process is also known as “electrical remodeling”. Genetic studies of AF have further identified mutations in ion channel genes (e.g., *KCNQ1, KCNH2, SCN5A, SCN10A*) that increase susceptibility to AF (4, 5). Atrial structural remodeling is primarily characterized by alterations in the ultrastructure of atrial myocytes and interstitial fibrosis. Persistent atrial electrical and structural remodeling, commonly observed in patients with AF, contributes to the development of a well-characterized form of atrial cardiomyopathy, which manifests through various clinical symptoms. The concept of atrial cardiomyopathy was formally introduced in the 2016 Expert Consensus Statement, which categorized it into four distinct subtypes based on pathophysiological mechanisms (EHRAS classification), with cardiomyocyte fibrosis being the predominant pathological hallmark. Importantly, the progression of atrial cardiomyopathy facilitates the maintenance of AF, while sustained AF in turn aggravates atrial remodeling and impairs mechanical function, thereby establishing a self-perpetuating vicious cycle (6). Although many pathophysiological aspects of electrical and structural remodeling—particularly ion channel dysfunction and atrial fibrosis—have been elucidated, there remains a lack of specific AF-related ion channel gene biomarkers and no definitively effective clinical targets for atrial fibrosis. Therefore, a more in-depth understanding of these pathophysiological mechanisms is urgently needed to break the vicious cycle of AF progression and to identify therapeutic breakthroughs.

It is well known that AF arises from the combined influences of genetic and environmental factors. High-throughput sequencing technologies, such as genome-wide association studies (GWAS) and whole-exome sequencing (WES), have advanced our understanding of multiple genetic susceptibility variants in AF (7, 8). Notably, although identifying pathogenic AF genes from peripheral blood and tissue-level analyses has provided valuable insights into the pathophysiology of AF, a systematic evaluation of the ion channel gene set in AF, as well as in-depth knowledge of cell-to-cell relationships—particularly involving atrial fibroblasts—remains relatively limited.

Recent cell atlas research on the normal human heart has shown that the heart primarily consists of cardiomyocytes, smooth muscle cells, endothelial cells, and fibroblasts (9). Nevertheless, at the single-cell level, our understanding of the specific gene alterations, regulatory processes, and cell–cell interaction genes in atrial fibroblasts—which are key cells driving structural remodeling in AF—is insufficient. In particular, single-cell RNA sequencing (scRNA-seq) has recently been broadly applied in scientific research and is well suited for identifying specific pathogenic cell types and genes involved in disease processes (10). Therefore, identifying the critical pathogenic fibroblasts in AF and elucidating the differences and interactions between these fibroblasts and other cell types could greatly enhance our understanding of AF pathophysiology and catalyze the development of targeted therapeutic.

In our study, single-cell sequencing data from atrial fibroblasts were utilized in conjunction with pseudotime trajectory analysis, cell–cell interaction analysis, and enrichment analysis to thoroughly investigate cellular distribution, developmental trajectories, intercellular interactions, and the biological functions and pathways associated with specific cell populations in AF. These findings were then comprehensively compared between AF and SR. Additionally, we innovatively integrated single-cell data from AF with microarray expression profiles, enabling the identification of ion channel signature genes in AF. Finally, the DGIDB database was employed to predict the druggability of key gene subpopulations implicated in AF-associated structural remodeling, as well as the identified ion channel signature genes. This approach provides critical insights into potentially druggable cells and genes in AF.

## Materials and methods

### Single-cell data processing

To investigate the structural remodeling of AF at the single-cell level, the single-cell dataset (GSE148506) was obtained from the GEO database (11). This dataset comprises freshly isolated atrial cardiofibroblasts from four AF patients and four control patients (sinus rhythm, SR) and is based on SmartSeq2 sequencing technology and the GPL18573 Illumina NextSeq 500 platform. Analyses were performed using the “Seurat” R package (version 4.2.0) (12). First, raw data were subjected to quality control and filtering under the following criteria: each gene had to be expressed in at least 3 cells, the number of genes expressed per cell was between 200 and 10,000 (200 < nFeature_RNA < 10,000), and both mitochondrial and ribosomal content were less than 20%. After filtering, the preprocessed data were normalized and used to identify the top 2,000 highly variable genes via the “LogNormalize”, “ScaleData”, and “vst” functions. These 2,000 highly variable genes were then used for further dimensionality reduction (“PCA”) to identify principal components (PCs). Clusters were determined using the “FindNeighbors” and “FindClusters” functions (resolution = 0.8), and 20 PCs were selected for t-SNE analysis. Significant marker genes in different clusters [The filtering criteria were: log fold change (logFC) >0.5 and adjusted *p*-value (adjPval) <0.05] were identified with the “FindAllMarkers” function. Finally, different cell types were characterized based on the expression of cluster-specific marker genes, with auxiliary annotations from the “SingleR” package and by consulting the annotation results of the original study's authors.

### Cell pseudotime analysis

Cellular pseudotime and trajectory analyses were employed to investigate the differentiation pathways of various annotated cell subgroups, as well as the genes associated with different trajectory patterns, thereby elucidating the molecular mechanisms underlying the structural remodeling of AF (13). The “Monocle” package was used to cluster the cells, and the “DifferentialGeneTest” function identified differentially expressed genes among different cell types. Subsequently, the “DDRTree” function (minSpanningTree; num_clusters = 4) was applied to infer pseudotime trajectories between different cell subtypes and to generate heatmaps and trajectory maps depicting potential developmental relationships among these cell populations.

### Cell-cell interaction analysis

To investigate the interactions among different cell types during the structural remodeling of AF, a cell–cell communication analysis was performed using the “CellChat” package in R (14). This package contains a ligand–receptor library that enables the simulation of cell–cell interactions based on specific ligand–receptor pairs. Depending on the ligands and receptors expressed by individual cells, the package defines those cells as corresponding signal senders or receivers.

### Biological function and pathway enrichment analysis

Two complementary approaches were used in this study to elucidate the biological functions and signaling pathways associated with the gene sets of interest. The first involved conducting Gene Ontology (GO) and Kyoto Encyclopedia of Genes and Genomes (KEGG) enrichment analyses using the “clusterProfiler” and “enrichplot” packages in R (15). The second approach utilized three gene collections from the Molecular Signatures Database (MsigDB)—the “C5 collection (GO:BP gene set),” the “C2 collection (KEGG gene set),” and the “H collection (hallmark gene set)”—to perform Gene Set Enrichment Analysis (GSEA) (16, 17). Enrichment results with *P*-value < 0.05 were then visualized using the “ggplot2” and “ggpubr” packages.

### Collection and pre-processing of microarray expression profiling data and ion channel-associated genes

To further explore key genes involved in AF-related electrical remodeling, the microarray expression profile datasets GSE79768 (18) and GSE115574 (19) were downloaded from the GEO database. Both datasets are based on the GPL570 sequencing platform, with GSE79768 comprising atrial tissue specimens from 13 patients with permanent AF and 13 patients with SR, and GSE115574 containing atrial tissue from 31 patients with permanent AF and 28 patients with SR. Subsequently, the two datasets were individually probe-annotated and normalized. The “sva” package in R was then used to merge the preprocessed datasets and remove batch effects. The “ComBat” function from the “sva” package is widely used to correct batch effects in high-throughput datasets. Based on systematic evaluations demonstrating its superior performance over other methods, we selected “ComBat” to eliminate batch effects between the two datasets. Furthermore, ion channel–related genes were identified based on an extensive literature review. A detailed list of these genes is provided in Supplementary Table S1 for subsequent analyses.

### Differential analysis and machine-learning algorithms for screening ion channel signature genes in AF

Using the ion channel gene collection, we extracted ion channel gene expression data from the GSE79768 dataset and performed a differential analysis with the “limma” package (20). The screening criteria of DEGs were identified using a log fold change >1 and adjusted *p*-value < 0.05. Next, two machine-learning algorithms—“LASSO” and “SVM-RFE”—were employed to further refine the differentially expressed genes (DEGs) and identify key ion channel signature genes in AF. The LASSO algorithm, implemented through the “glmnet” package in R, uses tenfold cross-validation to select genes strongly associated with AF. The SVM-RFE algorithm (recursive feature elimination), based on the “e1071” package in R, similarly identifies genes showing significant differences between AF and SR. The same method was used to identify ion channel DEGs in the GSE115574 dataset. Finally, the genes identified by the machine-learning algorithms in GSE79768 were intersected with the DEGs discovered in GSE115574 to reduce errors introduced by using a single dataset and to improve the accuracy of the identified genes.

### Evaluation of ion channel signature genes in AF

Based on the two datasets after merging and removing batch effects, boxplots were used to demonstrate the difference in expression levels of ion channel signature genes in AF and SR. the ROC curve and area under the curve (AUC) were used to assess the power of signature genes to discriminate in AF and SR.

### Drug enrichment analysis

Using the DSigDB database (DSigDB | Tan Lab), a drug–gene interaction file was obtained (see Supplementary Table S2). Drug enrichment analyses were then performed on EF cell subgroups (Genes selected with logFC >2) and on the ion channel genes *ANO1* and *GRIK2*, utilizing the “clusterProfiler” and “enrichplot” packages in R. The results were visualized through a gene concept network diagram, and a corrected *P*-value < 0.05 was considered statistically significant.

## Results

### Single-cell analysis identified key cell clusters

**Figure 1:**
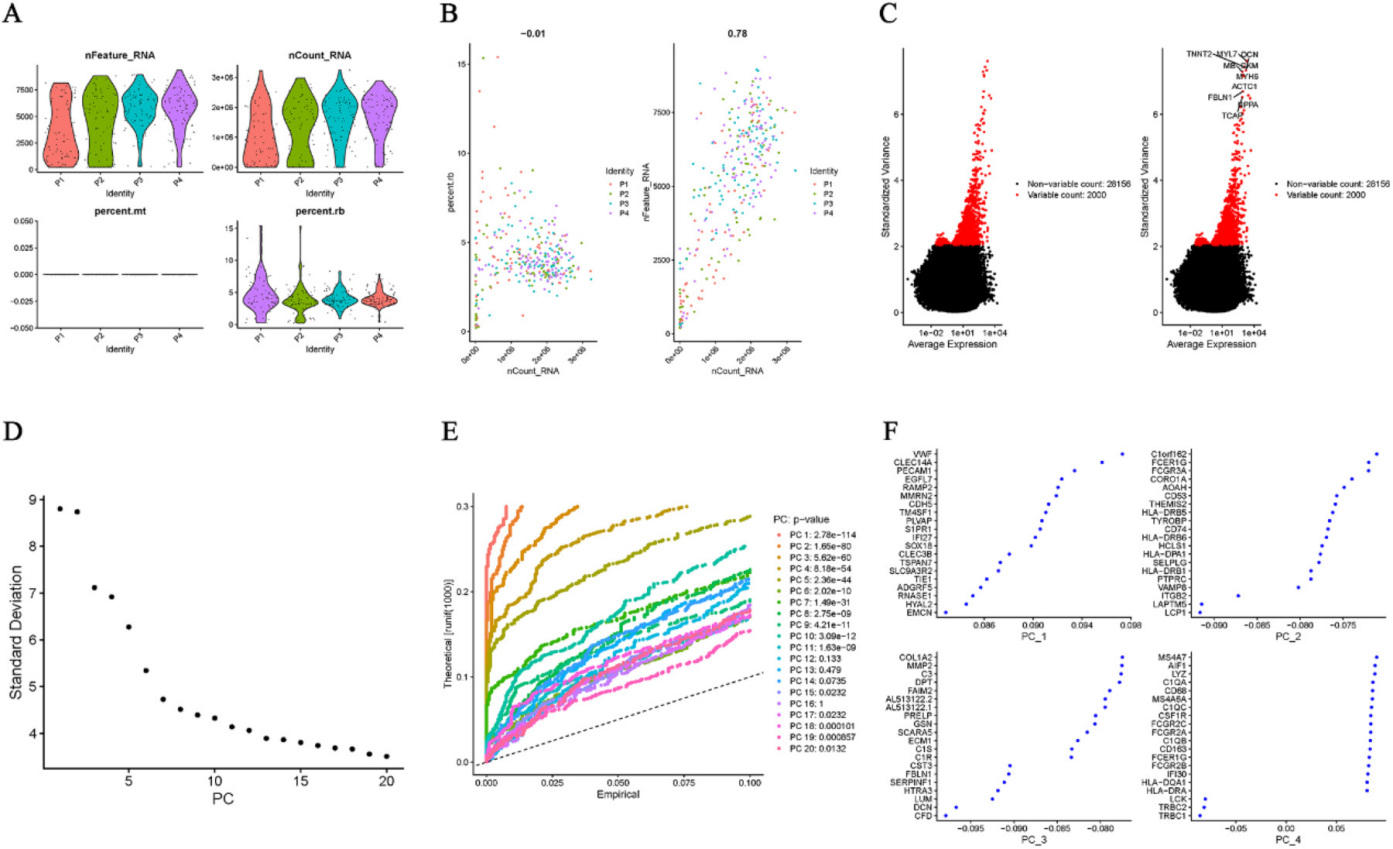
Quality control, HVG selection, and PCA. **(A)** QC metrics (nFeature_RNA, nCount_RNA, and percent.rb). **(B)** Correlation between nCount_RNA and nFeature_RNA (*r* = 0.78). **(C)** Top 2000 HVGs. **(D)** ElbowPlot for determining the number of significant PCs. **(E)** JackStrawPlot identifying 20 significant PCs (resolution = 0.8). **(F)** Top four PCs with their 20 HVGs each.

The expression characteristics of the single-cell dataset, following quality control and filtering, are presented in Figure 1A. “nCount_RNA” is not related to “percent.rb,” but shows a positive correlation with “nFeature_RNA” (*r* = 0.76; Figure 1B). The top 2,000 highly variable genes (HVGs) are displayed in Figure 1C. Based on the ElbowPlot (Figure 1D**)** and the “FindNeighbors” function (resolution = 0.8), 20 principal components (PCs) were identified through PCA, as shown in the JackStrawPlot (Figure 1E). The top four PCs, each with 20 highly variable genes, are illustrated in Figure 1F.

### Annotation and enrichment analysis of different cell types

**Figure 2:**
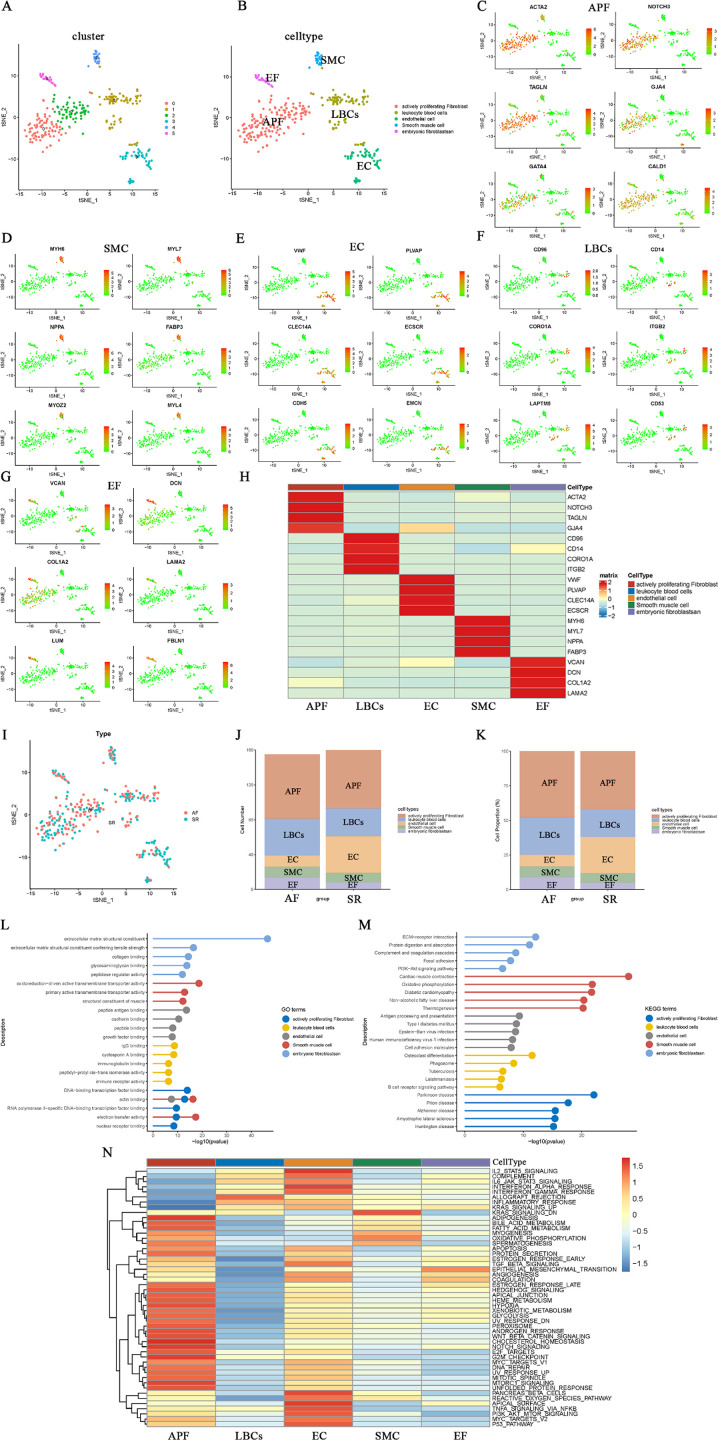
Clustering, annotation, and enrichment analyses of single-cell data. **(A)** t-SNE plot of six clusters derived from 20 principal components. **(B)** Cell-type annotation (five types) based on marker genes and the singleR package. **(C–G)** Marker gene expression in APF **(C)**, SMC **(D)**, EC **(E)**, LBCs **(F)**, and EF **(G**,**H)** Heatmap of the top four marker genes for each cell type. **(I**-**K)** Cell distribution, counts, and proportions in AF vs. SR for each cell type. **(L–N)** GO, KEGG, and GSEA (“HALLMARK”) enrichment results for the five cell types.

Subsequently, the 20 PCs were clustered into six cell subgroups using t-distributed stochastic neighbor embedding (T-SEN) (Figure 2A). These six subgroups were then annotated into five cell types based on marker gene expression and the auxiliary cell annotation provided by the “singleR” package (Figure 2B). Specifically, the largest cell type—actively proliferating fibroblasts (APF)—showed high expression of ACTA2, NOTCH3, TAGLN, GJA4, GATA4, and CALD1 in clusters 0 and 2 (Figure 2C). In contrast, cluster 4, which displayed myosin-enriched marker genes (MYH6, MYL7, NPPA, FABP3, MYOZ2, and MYL4), was annotated as smooth muscle cells (SMC) characterized by contractile function (Figure 2D). Cluster 3 consisted of endothelial cells (EC) expressing VWF, PLVAP, CLEC14A, ECSCR, CDH5, and EMCN (Figure 2E). Cluster 1 comprised leukocyte blood cells (LBCs) with CD96 and CD14 as marker genes (Figure 2F). Cluster 5 was defined as embryonic fibroblasts (EF)—ACTA2-negative cells expressing VCAN, DCN, COL1A2, LAMA2, LUM, and FBLN1—situated between fibroblasts and myofibroblasts (Figure 2G). Heatmaps illustrating the top four marker genes for each cell type are shown in Figure 2H. All DEGs for these five cell types are provided in Supplementary Table S3. The distribution, cell count, and cell proportions of AF and SR for these five cell types are shown in Figures 2I–K, revealing that AF features higher proportions of APF, LBCs, and EF, whereas SR has a greater proportion of EC. GO, KEGG, and GSEA (“HALLMARK” gene sets) enrichment analyses were performed on the DEGs of each cell type; the results are depicted in Figures 2L–N; Supplementary Table S4.

The GO biological functions of EF are primarily enriched in extracellular matrix structural constituents and collagen binding (Figure 2L), while the KEGG pathways are chiefly enriched in ECM–receptor interactions and protein digestion and absorption (Figure 2M). The GO biological functions and KEGG pathways of SMC are mainly enriched in actin binding, structural muscle constituents, cardiac muscle contraction, oxidative phosphorylation, and diabetic cardiomyopathy. APF is predominantly enriched in DNA-binding transcription factor binding, actin binding, electron transfer activity, and neurological diseases closely related to fibrosis, including Parkinson's disease, prion disease, and Alzheimer's disease. LBCs primarily participate in inflammatory and immune responses, such as IgG binding, immunoglobulin binding, immune receptor activity, and phagosome-related processes. EC are largely involved in peptide antigen binding, growth factor binding, antigen processing and presentation, and cell adhesion molecules. In the GSEA (“HALLMARK” gene set) enrichment analysis (Figure 2N), EF exhibited the highest score for epithelial_mesenchymal_transition. Additionally, EC is largely enriched in tissue-surface–related terms (e.g., apical_surface and pancreas_beta_cells), and is also involved in inflammatory and immune responses similar to LBCs, including IL2_STAT5_signaling, IL6_JAK_STAT3_signaling, and interferon_alpha_response. These functions and pathways of EC and LBCs differ markedly from those of APF, which is mainly enriched in metabolic responses, growth, and hypoxic stress processes, such as heme_metabolism, glycolysis, hypoxia, notch_signaling, and unfolded_protein_responses.

### Trajectory analysis of different cell types

**Figure 3:**
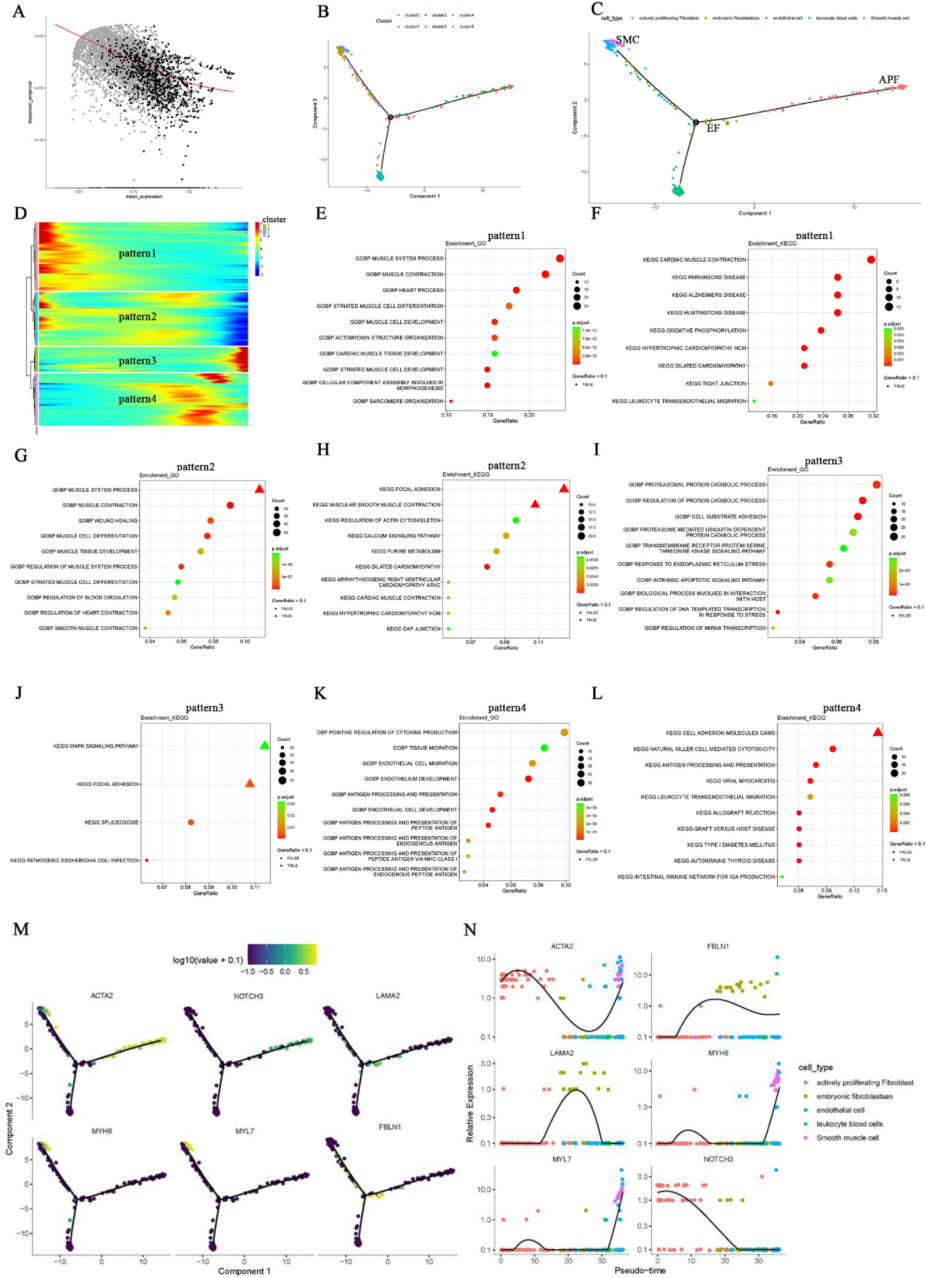
Pseudotime analysis of fibroblast developmental trajectory in AF. **(A)** Differential genes identified by the Molocole “differentialGeneTest” function and reduced by the “DDRTree” function. **(B**,**C)** Distribution of cell subgroups along the pseudotime trajectory. **(D)** Heatmap showing four relative expression patterns of marker genes along the trajectories. **(E–L)** GO and KEGG enrichment results for the four relative expression patterns. **(M)** Distribution of EF, APF, and SMC marker genes along the trajectory. **(N)** Pseudotime plots of marker gene expression for different cell types.

In order to elucidate the developmental trajectories of fibroblasts involved in AF-related structural remodeling, we conducted a pseudotime analysis of the annotated cell subgroups using “Monocle.” The “differentialGeneTest” function in Monocle identified differentially expressed genes (DEGs) across cell types for further dimensionality reduction by the subsequent “DDRTree” function (Figure 3A). Figures 3B,C illustrate the distribution of the different clusters and cell subgroups on the trajectory map, and the heatmap in Figure 3D shows four relative expression patterns for the marker genes of these cell types along the inferred trajectories. GO and KEGG enrichment analyses were then performed on each of the four relative expression patterns (Figures 3E–L; Supplementary Table S5). The findings indicate two predominant trajectory patterns. In one of them, GO (BP) is primarily enriched in muscle system processes and muscle cell differentiation (Figures 3E,G), whereas KEGG enrichment mainly involves muscle contraction, cardiomyopathy, and pathways related to neurological diseases (Figures 3F,H). We speculate that this pattern encompasses EF, APF, and SMC. In the other trajectory pattern, GO (BP) is predominantly enriched in positive regulation of cytokine production, antigen processing and presentation, and endothelial cell migration (Figures 3I,K**)**. Correspondingly, KEGG enrichment focuses on cell adhesion molecules, NK cell–mediated cytotoxicity, antigen processing and presentation, and MAPK signaling pathways (Figures 3J,L). We infer that EC and LBCs likely fall into this second pattern. We subsequently analyzed the distribution of EF, APF, and SMC marker genes within the trajectory plots (Figure 3M) and examined their relative expression in different cell types across pseudotime (Figure 3N). Notably, EF (cluster 5) appear capable of developing into either APF (clusters 0 and 2) or SMC subgroups exhibiting contractile function (cluster 4). This cardiac fibroblast developmental trajectory may be critical for understanding structural remodeling in AF.

### Analysis of cell-cell interaction networks for structural remodeling in AF

**Figure 4:**
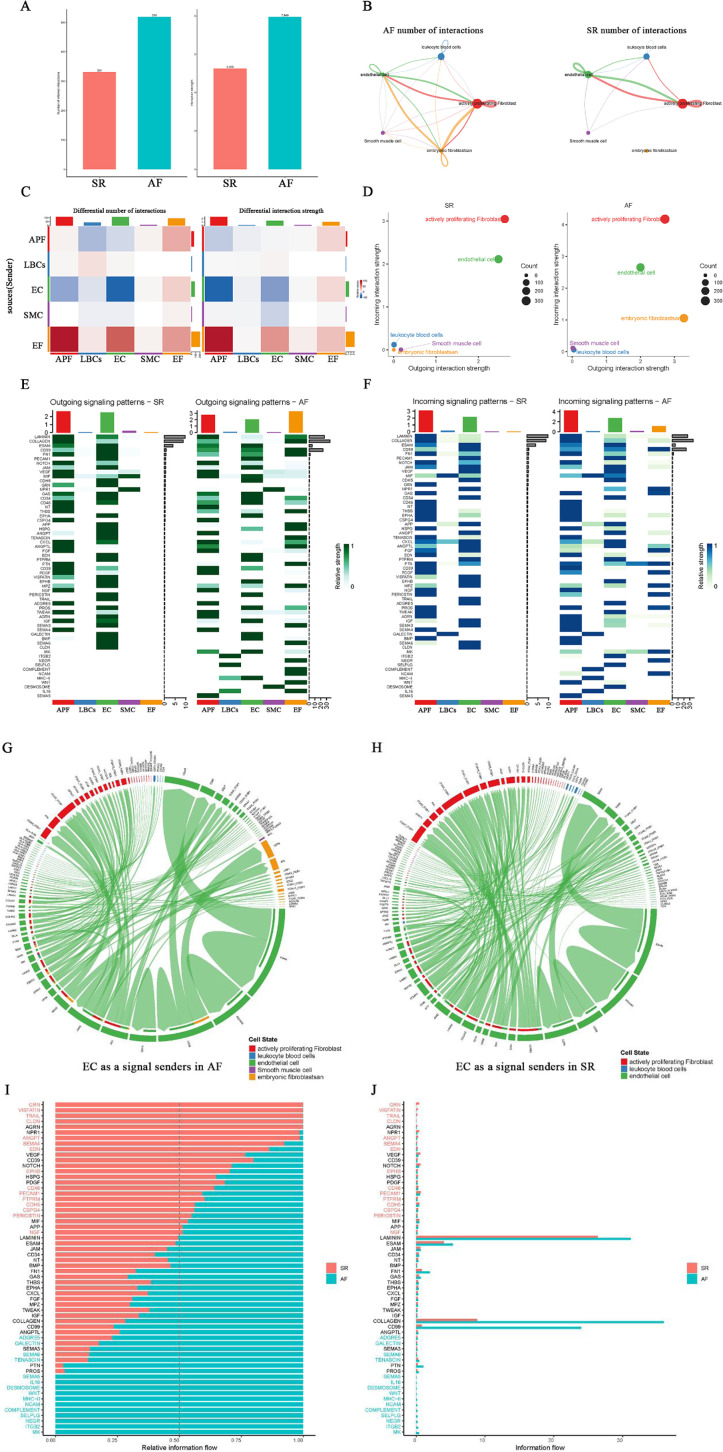
Cell-cell interaction analysis in AF and SR. **(A)** Comparison of the number and strength of cell interactions between AF and SR. **(B)** Network plot showing cellular interactions, with more interactions between EF, APF, EC, LBCs, and SMC in AF, particularly EF interactions. **(C)** Cell interaction network heatmaps showing differences between AF and SR. **(D)** Two-dimensional scatter plots of incoming and outgoing signal interaction strengths, highlighting changes in APF, EF, and EC signals in AF. **(E**,**F)** Overall signaling patterns and receptor signaling differences, including weakened NPR1 signaling in EC and the absence of specific signals in EC outgoing patterns in AF. **(G**,**H)** Chord diagrams showing EC as signal senders in AF and SR. **(I**,**J)** Histograms comparing relative and absolute information flow in AF and SR, highlighting signals unique to AF and SR.

To investigate the interactions among cell types involved in AF-related structural remodeling, a cell–cell interaction analysis was performed. The results showed that both the number and strength of cell interactions were significantly higher in AF than in SR (Figure 4A). Network plots of cellular interactions revealed more interactions among EF, APF, EC, LBCs, and SMC in AF; in particular, EF displayed interactions with other cell types in AF but almost none in SR (Figure 4B). The cell interaction network heatmaps further illustrate the differences in the number and strength of cell–cell interactions between AF and SR (Figure 4C). Subsequently, we compared outgoing, incoming, and overall signaling in AF and SR (Figures 4E,F; Supplementary Figure S1). Two-dimensional scatter plots of incoming and outgoing interaction strength clearly indicate that the enhanced incoming signals for APF, EF, and EC, along with the enhanced outgoing signals from EF in AF, coincide with weakened outgoing signals from EC (Figure 4D). An examination of the overall signaling patterns reveals extensive cell–cell interaction signals activated in EF in AF (e.g., *LAMININ, COLLAGEN, CD99, APP, PTN, TENASCIN, and SEMA3*) that are absent in SR. In addition, *MK, ITGB2, NEGR, SELPLG, COMPLEMENT, NCAM, MHC-II, WNT, DESMOSOME, IL16*, and *SEMA5* signals were only present in AF and not in SR (Figures 4E,F; Supplementary Figure S1). Regarding outgoing signaling patterns, *GRN, APP, HSPG, ANGPT, VISFATIN, TRAIL, SEMA3, AGRN, SEMA6*, and *CLDN* signals from EC were absent in AF, although they were normally present in SR (Figure 4E). Moreover, in the incoming signal patterns, the *NPR1* receptor for *NPPA* (encoding atrial natriuretic peptide, ANP, whose mutations can cause AF) in EC signaling appeared weakened in AF compared to SR (Figure 4F). Notably, NPR1 signaling was only observed in the outgoing signals of SMC (Figure 4E). Chord diagrams were then used to visualize all signals from EC in AF and SR (Figures 4G,H). Subsequently, we compared the proportion of relative information flow and the absolute number of information flows in AF and SR with bar plots (Figures 4I,J). Notably, the signals present only in SR originate exclusively from EC, whereas among the signals present only in AF are *MK, WNT, and SEMA5 in APF; ITGB2, SELPLG, COMPLEMENT, MHC-II,* and *IL16* in LBCs; *MK, ITGB2, SELPLG, and DESMOSOME* in EC; *DESMOSOME* in SMC; and *MK, NEGR, COMPLEMENT, NCAM, and WNT* in EF. Additionally, *LAMININ, COLLAGEN, and CD99* had the highest number of information flows, all activated in EF under AF but absent in EF under SR.

### Exploring the large number of activated ligand-receptor pairs signaling in EF of AF and in EC and SMC of SR

**Figure 5:**
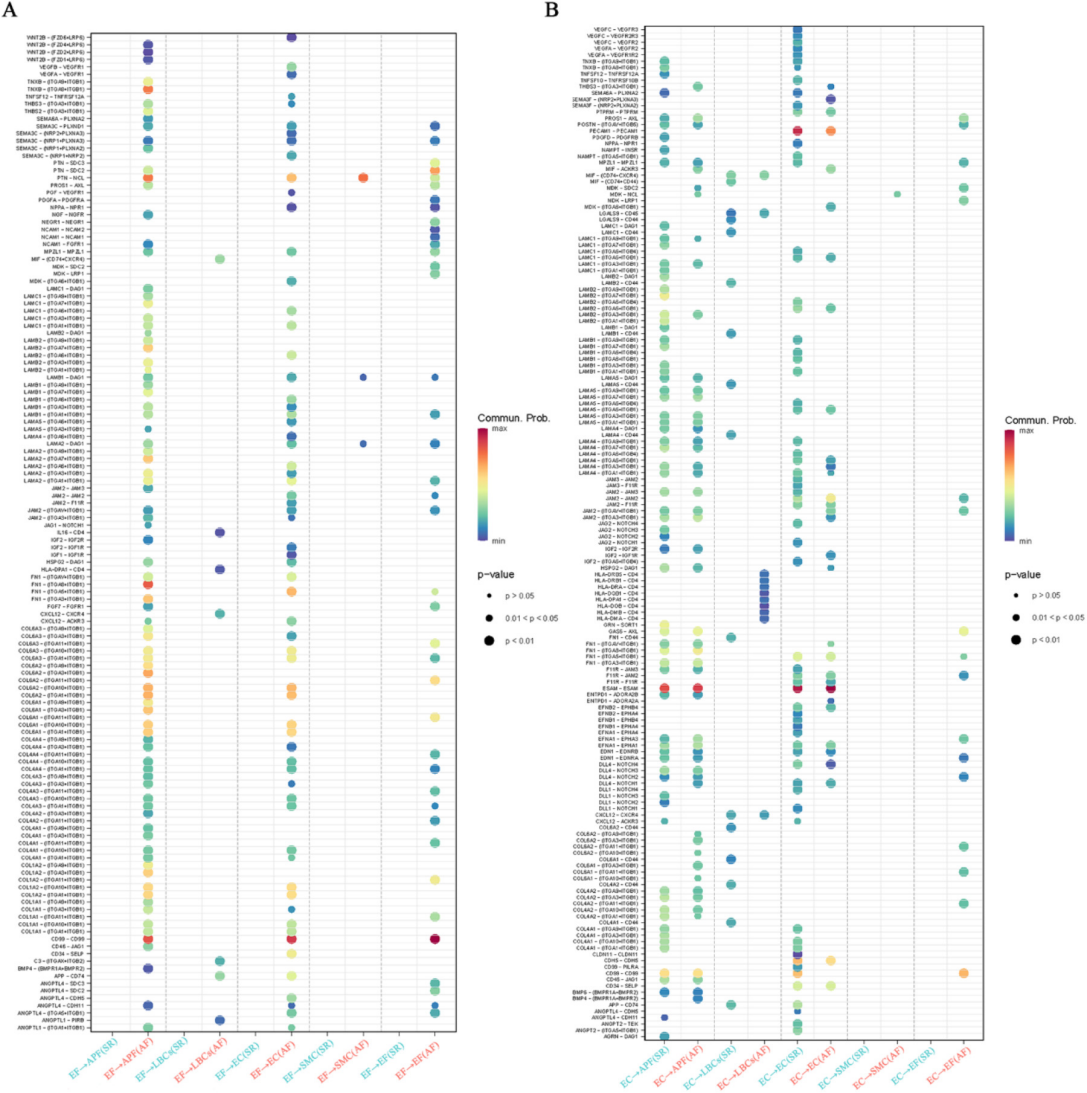
Ligand-receptor pair analysis of EF, EC, and SMC signaling in AF and SR. **(A)** Ligand-receptor pairs in EF as signal senders, highlighting interactions with APF, EC, SMC, and autocrine signals via PTN-NCL, CD99-CD99, and integrin receptor families (LAMININ, COLLAGEN, TNXB, FN1). **(B)** Ligand-receptor pairs in EC as signal senders, showing missing signals (VEGF-VEGFR, ANGPT-TEK) in AF and increased signals to LBCs (HLA-DRB-CD4) in AF.

Since all EF signaling is activated exclusively in AF, certain EC signals are present only in SR, and some SMC signals appear to be weakened in AF, we explored and compared the ligand–receptor pairs involving EF as both signal sender and receiver, as well as EC and SMC as signal senders in AF and SR. The results are illustrated with bubble plots (Figures 5A,B; Supplementary Figures S2, S3). Interestingly, when EF acts as a signal sender, it can transmit signals to APF, EC, SMC, and through autocrine pathways via PTN–NCL ligand–receptor pairs, and to APF, EC, and via autocrine pathways through CD99–CD99 ligand–receptor pairs. In addition, EF can bind to integrin receptor family members (ITGA*/ITGB*) via ligands such as LAMININ, COLLAGEN, TNXB, and FN1, thereby sending signals to APF, EC, and through autocrine pathways (Figure 5A).

When EC acts as a signal sender, we found that EC autocrine signals—VEGF–VEGFR, EFNB/A1–EPHB4, JAM–F11R/JAM, APP–CD74, and ANGPT–TEK—and secretory signals to APF (e.g., GRN–SORT1, AGRN–DAG1, JAG2/DLL1–NOTCH3) were missing in AF. In contrast, signals sent from EC to LBCs (e.g., HLA-DRB/DQB/DRA/DMA–CD4) were increased in AF (Figure 5B). When EF acts as a signal receiver, it can receive signals from APF, EC, and autocrine pathways via MPZL1–MPZL1, MDK–SDC2/LRP1, CD99–CD99, GAS6–AXL, and certain integrin family ligand–receptor pairs. Furthermore, EF as a signal receiver shows increased PDGF, MDK, and GAS6 signaling and reduced LAMININ signaling compared with EF as a signal sender (Supplementary Figure S2). In the outgoing signals from SMC, the NPPA/B–NPR1 ligand–receptor pair from SMC to EC was significantly weaker in AF than in SR (Supplementary Figure S3). Notably, in EF signaling activated in AF, members of the integrin receptor family (ITGA*/ITGB*) play a pivotal role by binding the ligands LAMININ and COLLAGEN—responsible for the largest number of information flows—to form receptor–ligand pairs.

### Characterization of LAMININ, COLLAGEN and MK signals in AF and GRN, AGRN and NPR1 signals in SR

**Figure 6:**
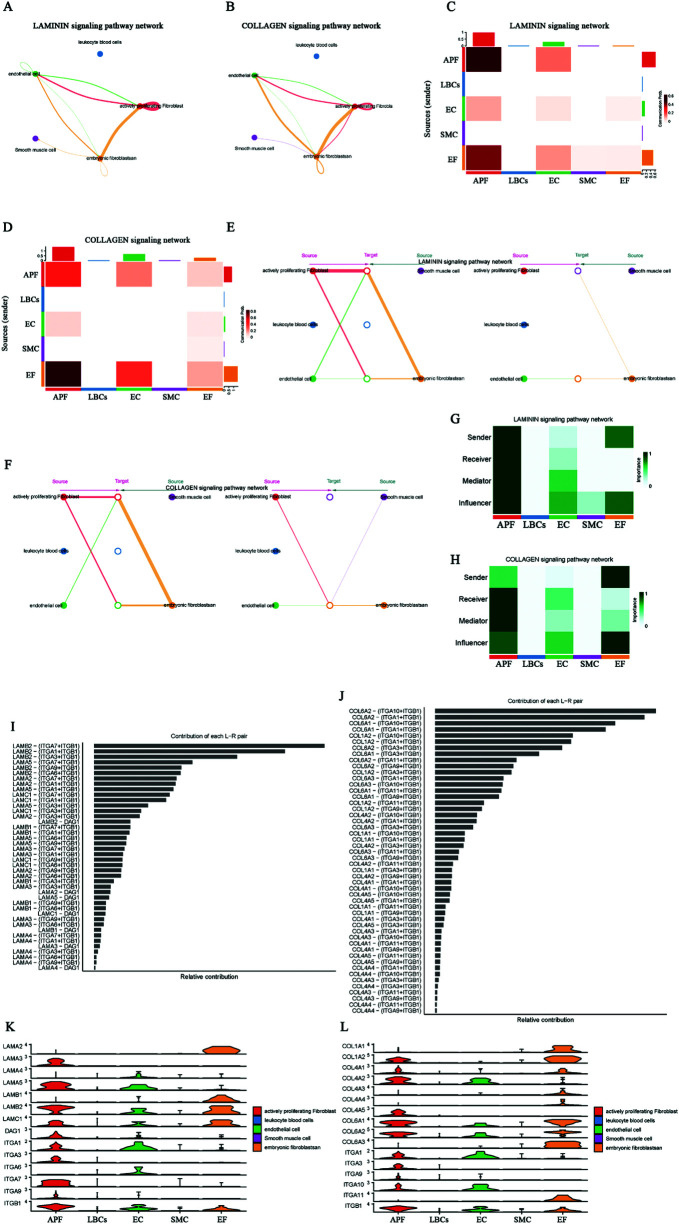
Characterization of LAMININ, COLLAGEN, GRN, AGRN, MK, and NPR1 signaling networks in AF and SR. **(A**,**C)** LAMININ signaling network, highlighting strong autocrine signals from APF and secretory signals from EF to APF. **(B**,**D)** COLLAGEN signaling network, showing stronger secretory signals from EF to APF compared to autocrine signals from APF. **(E)** EF as signal sender, with the strongest signal directed to APF. **(F)** EF cannot send signals to SMC in the COLLAGEN network. **(G)** EF as signal influencer in the LAMININ signaling network, with LAMB2-(ITGA3 + ITGB1) showing the highest contribution. **(I)** Expression of ligands and receptors in the LAMININ signaling network, with EF expressing ligands and APF/EC expressing more receptors. **(J)** COLLAGEN signaling network, where COL6A2-(ITGA10 + ITGB1) shows the highest contribution. **(K**,**L)** Expression of ligands and receptors in the LAMININ and COLLAGEN signaling network.

Since LAMININ and COLLAGEN signals are highly active among EF signals in AF, we further analyzed these two signals with substantial information flow. In addition, we highlight the GRN and AGRN signals sent by EC to APF in SR (absent in AF), the NPR1 signals sent by SMC to EC (weakened in AF), and MK signals exclusively present in the AF network. In the LAMININ signaling network, the two strongest signals are the autocrine signals from APF and those from EF to APF (Figures 6A,C). When EF acts as the signal source, it can send signals to APF, EC, SMC, and itself; the strongest signal is directed to APF (Figure 6E). Figure 6G shows that EF serves as both signal sender and influencer. LAMB2–(ITGA3 + ITGB1) contributes the most in this signaling network (Figure 6I). Figure 6K presents the expression levels of ligands and receptors in the LAMININ signaling network across five cell types, indicating that EF exclusively expresses the ligands, while APF and EC primarily express the receptors. In the COLLAGEN signaling network, EF shows a signaling pattern similar to that of LAMININ (Figure 6L). However, among the two strongest signals in the COLLAGEN network, the EF-to-APF signal is stronger than the autocrine APF signal (Figures 6B,D), and EF cannot send signals to SMC when serving as the signal source (Figure 6F). Additionally, COL6A2–(ITGA10 + ITGB1) provides the strongest signal contribution in the COLLAGEN signaling network (Figure 6J).

In the SR network of GRN and AGRN signaling, EC expresses the ligands, while APF expresses the receptors, allowing the transmission of GRN–SORT1 (Progranulin-Sortilin 1) and AGRN–DAG1 (Agrin-Dystroglycan 1) signals from EC to APF (Supplementary Figure S4). However, these EC-to-APF signals are absent in AF. In the MK (Midkine signaling) signaling network, the two strongest signals are those from EC and APF to EF, and both EC and APF also send signals to SMC. Consequently, EF and SMC mainly act as signal receivers, while EC and APF act as primary signal senders, with the MDK–NCL (Midkine–Nucleolin) ligand–receptor pair having the largest contribution (Supplementary Figure S5). The NPR1 signaling network shows that SMC signals are sent to EC, where NPPA–NPR1 (Atrial Natriuretic Peptide-Guanylate Cyclase A) exhibits a higher contribution (Supplementary Figure S6). These results clarify the ligand–receptor pairs involved in EF-activated signaling (LAMININ and COLLAGEN) in AF, the AF-specific MK signaling, the absence of certain EC outgoing signals (GRN and AGRN), and the weakened EC incoming signals (SMC's NPPA/B–NPR1) in AF. Collectively, these factors may be critical for AF-related structural remodeling.

### Differential analysis and functional enrichment analysis of ion channel-related genes in AF

**Figure 7:**
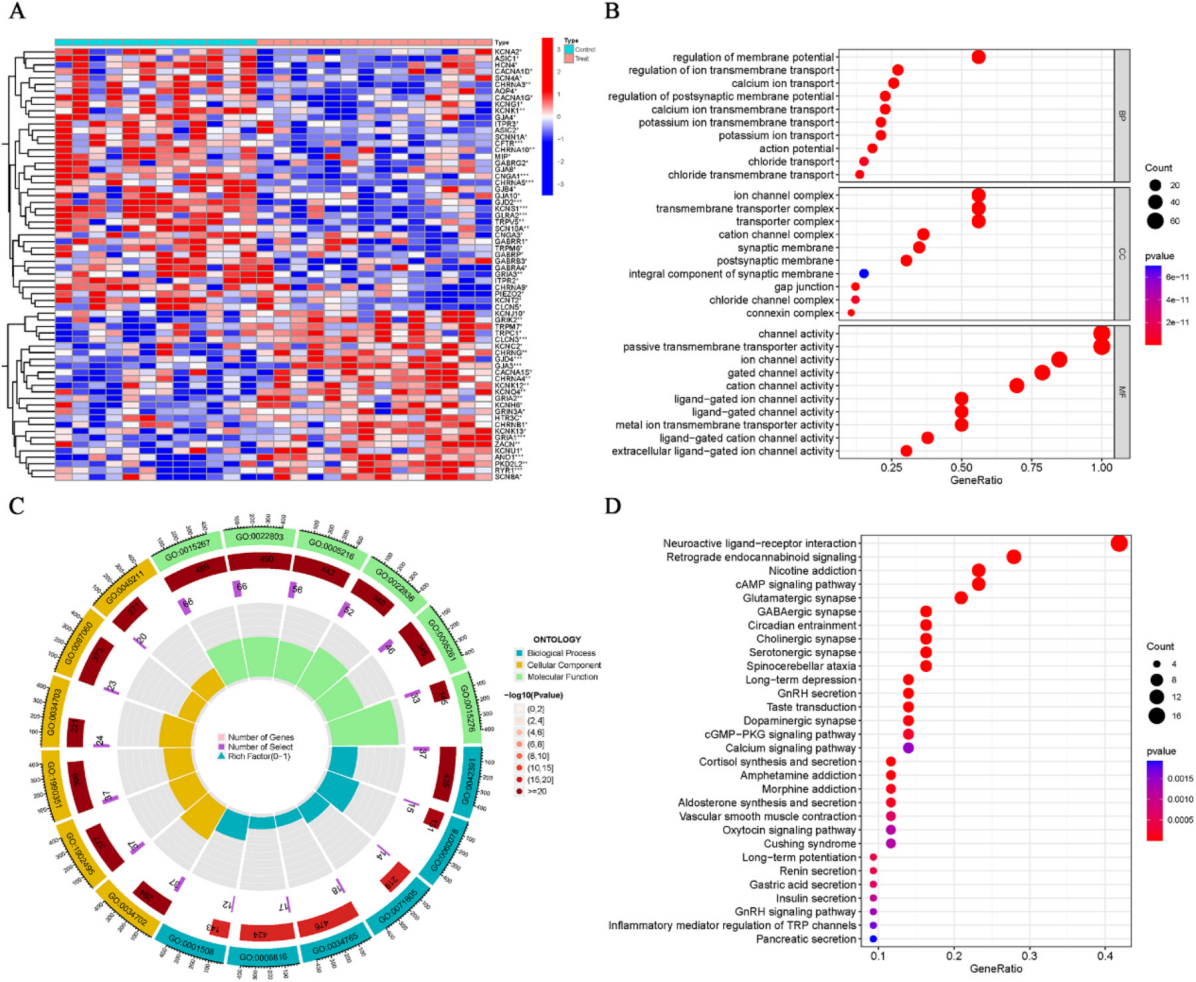
Differential expression and enrichment analysis of ion channel genes in AF. **(A)** Heatmap showing differential expression of ion channel genes between AF and SR groups (*P* < 0.05). **(B**,**C)** GO enrichment results for differentially expressed ion channel genes. **(D)** KEGG enrichment results.

The electrical remodeling of AF is closely linked to genes involved in ion channels. Hence, based on the ion channel gene set, we extracted the ion channel gene expression data from the normalized AF dataset (GSE79768) (Supplementary Table S6). Ion channel genes with differential expression between the AF and control groups were identified using a *P*-value threshold of <0.05, and the results of the differential analysis are displayed in a heatmap (Figure 7A; Supplementary Table S6). GO enrichment analysis of the differentially expressed genes revealed significant enrichment in terms such as “ion channel complex,” “channel activity,” and “regulation of membrane potential” (Figures 7B,C; Supplementary Table S6), whereas KEGG showed significant enrichment in pathways related to “neuroactive ligand–receptor interaction” (Figure 7D; Supplementary Table S6). These biological functions and pathways are strongly associated with ion channel genes.

### Machine learning identified ion channel signature genes in AF

**Figure 8:**
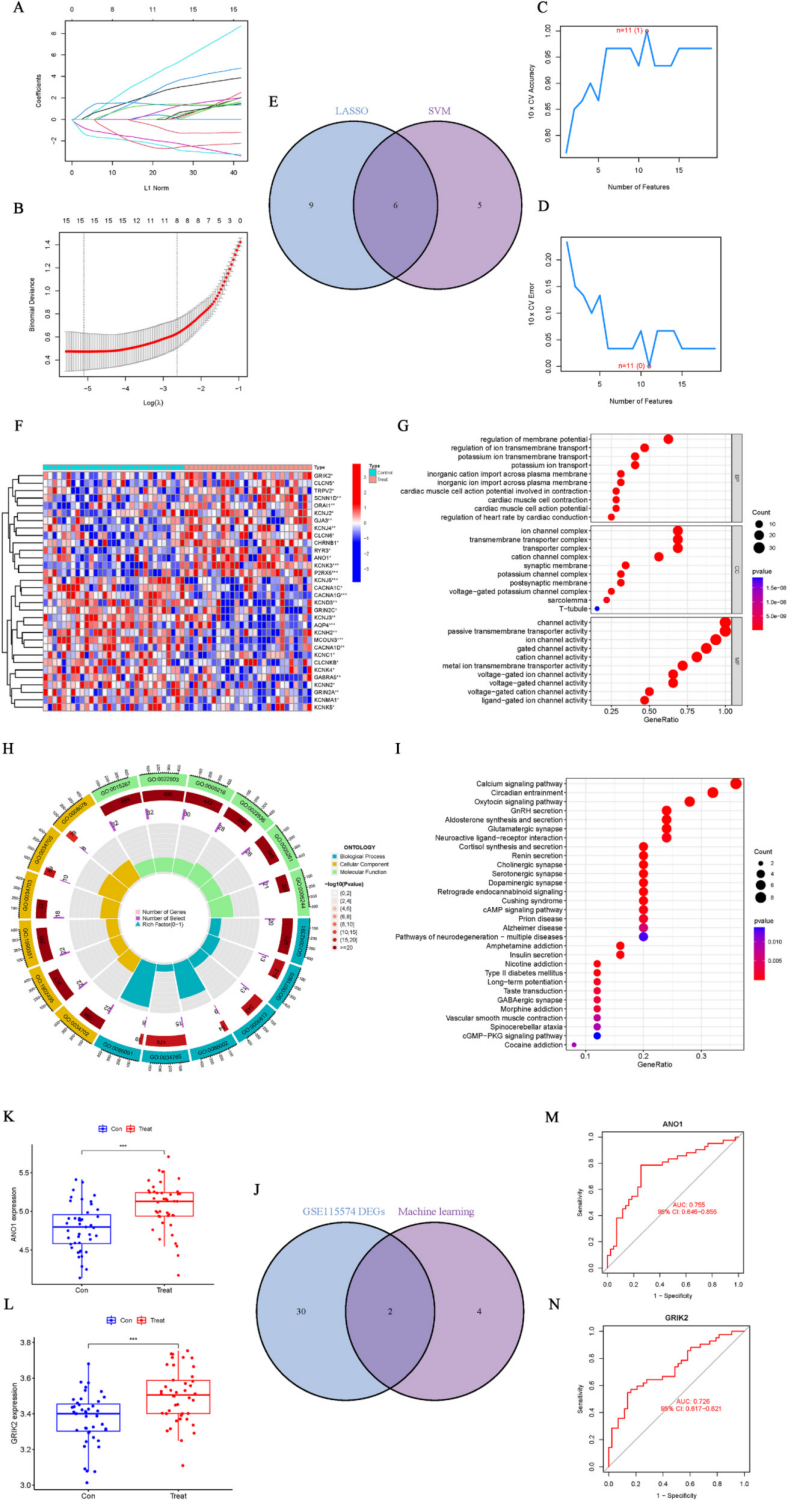
Identification and validation of ion channel signature genes in AF. **(A**,**B)** Ion channel genes identified by the “LASSO” algorithm. **(C**,**D)** Ion channel genes identified by the “SVM” algorithm. **(E)** Six overlapping ion channel signature genes identified by both algorithms. **(F)** Differential ion channel genes (GSE115574). **(G–I)** Enrichment analysis of differential ion channel genes, highlighting relevant biological functions and pathways. **(J)** Intersection of ion channel DEGs with the six overlapping genes identified by machine learning. **(K**,**L)** Boxplots showing significantly higher expression levels of *ANO1* and *GRIK2* in AF compared to SR (*p* < 0.001). **(M**,**N)** ROC curves for *ANO1* (AUC = 0.755) and *GRIK2* (AUC = 0.726) as diagnostic markers for AF.

Using both the “LASSO” and “SVM” machine learning algorithms for the ion channel DEGs enabled us to identify signature ion channel genes in AF. Fifteen ion channel genes were identified by LASSO (Figures 8A,B), and eleven by SVM (Figures 8C,D), resulting in six overlapping ion channel signature genes (Figure 8E). To minimize sample bias, the same methods were applied to the normalized AF dataset (GSE115574) to extract ion channel genes and perform differential and enrichment analyses of the DEGs. The differentially expressed ion channel genes are shown in Figure 8F; Supplementary Table S7, and the enrichment analysis results confirm close associations between these biological functions, signaling pathways, and ion channels (Figures 8G–I; Supplementary Table S7). Subsequently, the DEGs identified in GSE115574 were intersected with the six overlapping genes from the machine learning algorithms (Figure 8J), ultimately revealing *ANO1* and *GRIK2* as ion channel signature genes in AF. Box plots indicated that both *ANO1* and *GRIK2* had significantly higher expression in AF compared to the SR group (*p* < 0.001) (Figures 8K,L). The ROC curve showed that *ANO1* (AUC = 0.755) and *GRIK2* (AUC = 0.726) discriminated well between AF and SR (Figures 8M,N).

### The drug enrichment analysis of EF cell subgroups and the ion channel genes *ANO1* and *GRIK2*

**Figure 9:**
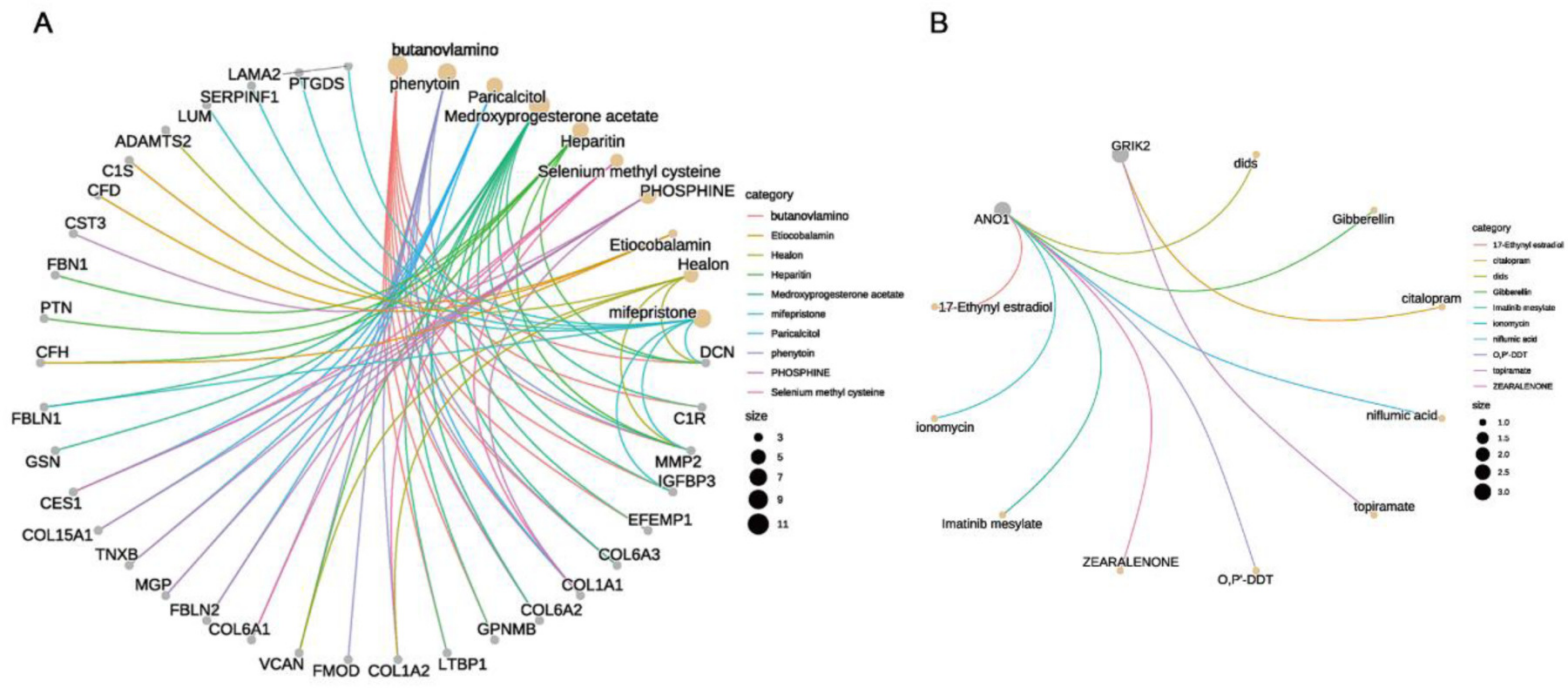
Druggability analysis of key cell subgroups and ion channel genes in AF. **(A)** Gene concept network diagram showing drugs significantly enriched in EF cell subgroups, including phenytoin, butanoylamino, and medroxyprogesterone acetate. **(B)** Druggability of key ion channel genes: *ANO1* linked to ionomycin, gibberellin, and dids; *GRIK2* linked to citalopram and topiramate.

After screening through machine learning methods and validating expression levels, *ANO1* and *GRIK2* were ultimately identified as signature ion channel genes associated with AF. Consequently, the druggability of key cell subgroups—specifically, EF cell subgroups, which may contribute to AF structural remodeling—as well as the key ion channel genes (*ANO1 and GRIK2*) involved in electrical remodeling, was further evaluated. The gene concept network analysis identified several drugs significantly enriched in the EF cell subgroups, including phenytoin, butanoylamino, medroxyprogesterone acetate, mifepristone, phosphine, and others (Figure 9A; Supplementary Table S8). Additionally, *ANO1* was associated with drugs such as ionomycin, gibberellin, and DIDS, while *GRIK2* was linked to citalopram and topiramate (Figure 9B; Supplementary Table S8). Notably, phenytoin, which was significantly enriched in the EF cell subgroups, has been previously confirmed to exhibit antiarrhythmic effects in prior studies.

## Discussion

AF has a complex etiology involving multiple aspects, such as structural remodeling and electrical remodeling. With respect to structural remodeling, numerous studies have examined processes including atrial fibrosis, myocyte hypertrophy and apoptosis, alterations in the extracellular matrix, autophagy, and inflammatory responses. Collectively referred to as “atrial remodeling,” these processes are closely associated with AF onset (21–23). However, a deeper understanding of structural changes in AF is still lacking, particularly regarding how cellular-level alterations initiate and sustain AF. Existing research largely focuses on changes in cardiac electrophysiology, with comparatively fewer investigations into cell-type heterogeneity and intercellular interactions. As single-cell analysis techniques have rapidly evolved, more studies have turned their attention to cell heterogeneity, particularly by leveraging single-cell RNA sequencing to elucidate the roles of different cell populations, thereby offering new perspectives on the cellular mechanisms underlying AF. In the domain of electrical remodeling, previous studies have shown that mutations or functional abnormalities in ion channel genes are closely linked to AF, including sodium channel genes *(SCN5A*), potassium channel genes (*KCNQ1, KCNH2, KCNE1, KCNE2*), calcium channel genes, hyperpolarization-activated cyclic nucleotide-gated channel genes (*HCN*), sodium–calcium exchanger genes (*NCX1*), and the titin gene (*TTN*) (24–29). Existing antiarrhythmic drugs are frequently associated with these ion channels. Nonetheless, although the potential roles of these ion channel genes in AF have garnered significant interest, research exploring the relationship between the human ion channel gene set and AF remains relatively scarce. Moreover, the identification of distinct ion channel gene biomarkers in AF has yet to be thoroughly addressed, and how alterations in other ion channels drive the onset and perpetuation of AF remains insufficiently revealed.

In the single-cell clustering and dimensionality reduction analysis, this study employed t-SNE clustering to divide 20 PCs into 6 subclusters. Based on marker gene expression and the “singleR” package for cell annotation, these subclusters were further categorized into 5 major cell types: APF, SMC, EC, LBCs, and EF. In this analysis, cluster 5 was defined as EF—a population of cells characterized by the absence of *ACTA2* expression and the presence of *VCAN, DCN, COL1A2, LAMA2, LUM*, and FBLN1. The biological features of these cells lie between those of quiescent fibroblasts and fully differentiated myofibroblasts. *ACTA2* (α-smooth muscle actin) is a classical marker of myofibroblasts; thus, the absence of *ACTA2* suggests that these EF cells do not exhibit the typical contractile phenotype of myofibroblasts. Instead, they represent a transitional or intermediate state, potentially with an embryonic-like phenotype, that is distinct from both resting fibroblasts and contractile myofibroblasts. GO, KEGG, and GSEA (HALLMARK gene set) enrichment analyses of DEGs in these 5 cell types supported each cell type's distinct biological functions and validated the accuracy of the clustering results. Additional analyses showed that the proportions of APF, LBCs, and EF cells increased in the AF group, whereas the EC proportion decreased. In contrast, the SR group exhibited a higher proportion of ECs, suggesting that these cells may play critical roles in the onset and progression of AF. This finding aligns with previous studies, which have indicated that fibroblast proliferation, imbalanced immune-inflammatory responses, and endothelial cell damage constitute key features of AF-related structural remodeling. Moreover, inflammation and immune pathways may serve as prerequisites for AF, subsequently leading to endothelial cell injury and atrial fibrosis and ultimately resulting in irreversible atrial remodeling. Damage to endothelial cells and hemodynamic disturbances further exacerbate thrombus formation in AF patients (30–33). Given the observed shifts in the distribution of different cell types between AF and SR, the study further investigated cell differentiation trajectories and intercellular interactions.

To investigate the developmental trajectories by which fibroblasts contribute to structural remodeling in AF, this study performed pseudotime analysis. The results revealed two primary trajectory patterns. One trajectory was primarily enriched in muscle system processes and muscle cell differentiation according to GO (BP) analysis, and in muscle contraction, cardiomyopathy, and neural disease pathways according to KEGG analysis; this trajectory is presumed to include EF, APF, and SMC cells. The other trajectory was enriched in the positive regulation of cytokine production, antigen processing and presentation, and endothelial cell migration (GO), and in cell adhesion molecules (CAMs), NK cell–mediated cytotoxicity, antigen processing, and MAPK signaling pathways (KEGG); it is thus speculated to involve EC and LBCs. Further examination of the distribution of marker genes for EF, APF, and SMC on the pseudotime trajectories, along with their relative expression levels, indicated that EF cells could develop along different paths into either the APF or a smooth muscle cell–like subtype (SMC). This suggests that EF cells may serve as key regulators of cardiac fibrosis and vascular smooth muscle remodeling. Previous research has shown that EF cells exhibit plasticity during cardiac injury repair and fibrosis, and that their differentiation may be influenced by factors such as inflammation, mechanical stress, and gene regulation (34, 35). On one hand, the differentiation of EF cells into APF—which have higher proliferative capacity—may promote cardiac fibrosis through excessive proliferation and the secretion of extracellular matrix proteins such as collagen and fibronectin, ultimately exacerbating atrial hardening and electrical conduction abnormalities that foster the onset and maintenance of AF (36). On the other hand, EF cells can also differentiate into SMC-like cells, potentially contributing to atrial vascular remodeling. Research indicates that, in chronic cardiovascular diseases, fibroblasts undergo transdifferentiation into smooth muscle–like cells through signaling pathways such as TGF-β, which may lead to abnormal vascular proliferation and dysfunction within the atria (37)**.** This study elucidates the developmental trajectories of EF cells in AF and offers new insights into the mechanism of structural remodeling in AF. Further work is needed to explore how EF cells selectively differentiate into APF or SMC under distinct pathological conditions, particularly the potential of key signaling pathways (e.g., TGF-β, Wnt, and Notch) as therapeutic targets. Investigations into treatment strategies targeting EF cells—such as regulating their differentiation via small-molecule drugs or gene editing technologies—may help mitigate pathological fibrosis. In summary, pseudotime analysis reveals the developmental trajectories of EF cells during AF-related structural remodeling, showing that EF cells can differentiate into either proliferative APF or contractile SMC. These findings not only deepen our understanding of AF-related structural remodeling mechanisms but also offer potential new targets for precision intervention in AF.

Subsequently, the study examined signal transmission and interactions among various cell types in AF and SR, with an emphasis on the roles of EF, EC, and SMC in the structural remodeling process. The results showed a marked increase in cell–cell signaling networks and interactions under AF compared to SR, particularly between EF cells and other cell types. This phenomenon suggests the presence of structural remodeling in cellular signaling during AF, notably involving EF cells. For instance, EF cells secrete multiple cytokines (e.g., LAMININ and COLLAGEN), thereby contributing to AF-related structural remodeling and potentially playing a critical role in the onset and maintenance of AF. Further analysis revealed that, in AF, the incoming signals received by APF, EF, and EC, as well as the outgoing signals from EF, were substantially enhanced, whereas the outgoing signals from EC were significantly reduced. Of particular note, *LAMININ* and COLLAGEN signaling were highly activated in AF and closely tied to EF cell functional transition and the fibrotic process. For example, LAMININ signaling, mediated by the LAMB2–(ITGA3 + ITGB1) ligand–receptor pair, exerts a key influence on EF cells and may impact cell adhesion and migration during atrial remodeling, as reported in other studies (38)**.** A similar pattern in the COLLAGEN signaling network further indicates that EF cells may play an essential role in the progression of atrial fibrosis (39, 40). In addition, the AF-specific MK and integrin receptor signaling pathways warrant attention, as they may contribute to the persistence of AF and cardiac remodeling, potentially forming a molecular foundation for establishing a fibrotic environment.

Regarding specific signaling pathways, EF was identified as a signal sender capable of transmitting extensive signals to APF, EC, and SMC cells through various ligand–receptor pairs (e.g., PTN–NCL, CD99–CD99). In contrast, the signaling activity of EC in AF underwent significant alterations, particularly in autocrine signals (e.g., VEGF–VEGFR, EFNB/A1–EPHB4), which were absent in AF, whereas signals from EC to LBCs were markedly elevated, suggesting that the role of EC in AF may have shifted with respect to interactions with other cell types. Notably, EC signal transmission in AF decreased considerably, especially in the absence of signals from the GRN–SORT1 and AGRN–DAG1 ligand–receptor pairs, indicating that EC may have lost its normal angiogenic and reparative functions in AF—an observation that may be closely tied to the pathology of AF (33, 41). Additionally, the NPPA–NPR1 signal from SMC to EC was notably weakened in AF, reflecting altered interactions between vascular smooth muscle cells and endothelial cells under AF conditions. The NPR1 signaling pathway plays a pivotal role in cardiovascular diseases such as atrial fibrosis and heart failure, and its attenuation in AF is strongly correlated with remodeling of cardiac structure and function (42, 43). In summary, the cell communication analysis revealed that AF onset and maintenance involve multiple signaling networks. In this study, several critical AF-associated pathways were highlighted, including extensive EF-mediated signals activated by ligands such as LAMININ and COLLAGEN, the AF-specific activation of MK (Midkine signaling) signaling, the absence of EC signals such as GRN–SORT1 (Progranulin-Sortilin 1) and AGRN–DAG1 (Agrin-Dystroglycan 1) in AF, and a marked reduction of the NPPA–NPR1 (Atrial Natriuretic Peptide-Guanylate Cyclase A) signal from SMC to EC. These signals are pivotal for AF onset and maintenance, particularly in terms of cell–matrix interactions, fibrotic processes, inflammatory and immune responses, and endothelial cell vascular remodeling. Future research could further validate the functions of these signaling pathways, particularly focusing on whether modulating ligand–receptor pairs can help suppress the structural remodeling observed in AF. In addition, exploring other factors that might influence these signaling pathways may open new therapeutic avenues for AF.

Ion channels play a crucial role in the electrophysiological remodeling of AF, where abnormalities in electrical activity are closely associated with dysfunction of ion channels. Research has shown that the onset of AF is linked to alterations in ion channel expression, particularly abnormalities in sodium, calcium, and potassium ion channels, which can obstruct the processes of cardiomyocyte depolarization and repolarization, ultimately leading to arrhythmias. Therefore, identifying and validating ion channel biomarker genes in AF may provide new molecular foundations and clinical targets for the prevention, diagnosis, and treatment of AF. Based on an ion channel gene set, this study extracted expression data of ion channel genes from the AF dataset (GSE79768) and identified significantly differentially expressed ion channel genes between the AF and control groups. Subsequently, by applying both LASSO and SVM machine learning algorithms, 6 overlapping ion channel signature genes were identified. To minimize sample bias, the same approach was applied to the GSE115574 dataset to extract ion channel genes, followed by differential analysis and enrichment analysis. *ANO1* and *GRIK2* were ultimately confirmed as ion channel signature genes in AF. The results indicated that *ANO1* and *GRIK2* were markedly upregulated in the AF group compared to the SR group. ROC curve analysis demonstrated that *ANO1* (AUC = 0.755) and *GRIK2* (AUC = 0.726) possess good discriminative power in distinguishing AF from SR. GO and KEGG enrichment analyses revealed that the differentially expressed ion channel genes were significantly enriched in key functions such as “ion channel complex,” “channel activity,” “regulation of membrane potential,” and the neuroactive ligand–receptor interaction pathway, which are closely related to the electrophysiological characteristics of AF (44).

Notably, this study employed two commonly used machine learning algorithms—LASSO and SVM—to screen for ion channel genes. LASSO incorporates an L1 regularization term that effectively reduces model complexity and identifies genes with strong predictive contributions. SVM, on the other hand, constructs a hyperplane to maximize the inter-class margin, making it well suited for feature selection in high-dimensional, small-sample datasets. Compared with traditional biostatistical methods, these two machine learning approaches can handle higher-dimensional data and detect potential nonlinear relationships, thereby providing robust tools for discovering novel disease marker genes (45). In this study, the two algorithms complemented each other, and comparing their outcomes yielded a relatively stable set of candidate genes. Ultimately, *ANO1* and *GRIK2* were identified as ion channel signature genes and biomarkers for AF, both of which were significantly upregulated in the AF group compared with the SR group. *ANO1* is a calcium-activated chloride channel whose cardiac expression is closely linked to the regulation of ion flux. Previous research has shown that increased *ANO1* expression can raise the density of calcium-activated chloride channels in ischemic hearts, causing arrhythmias triggered by ischemia (46). In the present study, the elevated expression of *ANO1* in AF may induce electrophysiological remodeling through its influence on cardiac electrical activity. *GRIK2* belongs to the glutamate receptor family, encoding the kainate receptor—also known as a glutamate-gated cation channel—which is predominantly found in the nervous system. Mutations in *GRIK2* underlie various neurodevelopmental disorders (47). Studies have indicated that these gated cation channels also contribute to heart rate and rhythm regulation, thereby affecting arrhythmogenesis (48, 49). However, research specifically focusing on the relationship between *GRIK2* and arrhythmias or AF remains limited. These findings suggest that *ANO1* and *GRIK2* may serve as important targets for the early diagnosis and treatment of AF.

In the study of electrical remodeling, a multi-layered and multi-dimensional analytical strategy was adopted to ensure the reliability and accuracy of the findings. First, differential expression of ion channel genes was repeatedly validated across multiple datasets. Second, differential expression analysis and enrichment analysis were combined to further elucidate the potential biological functions of ion channel genes in AF. Finally, the application of machine learning algorithms enabled the rational selection of ion channel signature genes with high clinical translational value. Considering sample size, data quality, and analytical rigor, the design of this study possesses notable scientific and clinical utility. Building on these results, the functions and specific mechanisms of *ANO1* and *GRIK2* in AF warrant further investigation. Future research could employ animal models or clinical trials to confirm the roles of these ion channel genes in AF, offering novel directions for precision AF treatment and potentially serving as targets for early diagnosis and therapy.

Lastly, this study further investigated the druggability of key cell subpopulations (EF) that may be involved in structural remodeling of AF, as well as the critical ion channel genes (*ANO1* and *GRIK2*) implicated in electrical remodeling. The analysis indicated that phenytoin sodium, butanoylamino, metopirone, mifepristone, and phosphoric were significantly enriched in EF cell subpopulations. Among these, phenytoin—widely recognized as a classic antiarrhythmic drug—has been shown by multiple studies to stabilize the cell membrane potential and reduce the excitability of cardiac myocytes, thereby mitigating AF (50–52). This suggests that EF cell subpopulations may represent a promising new therapeutic target for AF. Regarding *ANO1*'s druggability, the analysis revealed that ionomycin and other agents can influence *ANO1* function. As a potent and selective calcium ionophore, ionomycin may exert therapeutic effects by modulating apoptosis (53). However, other drugs strongly associated with *ANO1*, such as gibberellic acid and DIDS, remain understudied with respect to their regulatory effects on ion channels. Furthermore, this study found that *GRIK2* is associated with citalopram and topiramate. Citalopram, an antidepressant, has been shown to modulate neurocardiac interactions; previous reports indicate that it can affect cardiac conduction and repolarization, leading to symptomatic bradycardia and hypotension (54, 55). Topiramate is an antiepileptic agent, though whether it confers antiarrhythmic effects by acting on *GRIK2* expression remains uncertain. Notably, phenytoin sodium—another antiepileptic drug—is already recognized as a classic antiarrhythmic and was shown in this study to act on EF cells. An additional significant finding is the potential for drug repurposing. Medications such as citalopram and topiramate, originally developed for other conditions, show close correlations with ion channel genes in AF, suggesting a possible antiarrhythmic effect. Drug repurposing is a rapid and efficient approach to uncover novel indications for existing drugs, thereby reducing the time and cost associated with drug development (56). Despite revealing the potential roles of critical EF cell subpopulations and the ion channel signature genes *ANO1* and *GRIK2*, as well as proposing new therapeutic strategies via drug-targeting analysis, certain limitations remain. First, this study's datasets were predominantly drawn from public databases; therefore, additional clinical samples are needed to validate the clinical relevance of these genes and drugs. Second, the proposed drug targets must be verified through *in vitro* and *in vivo* experiments. Future investigations could evaluate the clinical therapeutic efficacy of targeting *ANO1* and *GRIK2* and integrate clinical data to provide further evidence for precision treatments of AF, thus offering new directions for AF drug-targeting research.

In conclusion, this study employed multiple analytical approaches—encompassing single-cell analysis, machine learning algorithms, and drug-targeting assessments—to investigate cell subpopulation distributions, developmental trajectories, and intercellular interactions in AF, as well as potential ion channel gene biomarkers in AF. Additionally, it identified key cell subpopulations and ion channel marker genes in AF and evaluated corresponding targetable drugs, providing novel insights into the structural and electrical remodeling mechanisms of AF. These findings offer promising directions for the precision treatment of AF and may serve as potential therapeutic targets in future AF research.

## Data Availability

The original contributions presented in the study are publicly available. This data can be found here: NCBI Gene Expression Omnibus (GEO); Accession Numbers: GSE148506, GSE79768, and GSE115574 (https://www.ncbi.nlm.nih.gov/geo/).

## Supplementary material

Supplementary Table S1 List of ion channel–related genes identified through literature review for downstream analyses.

Supplementary Table S2 Drug–gene interaction dataset obtained from the DSigDB database.

Supplementary Table S3 Differentially expressed genes (DEGs) across the five studied cell types.

Supplementary Table S4 Results of GO, KEGG, and GSEA (“HALLMARK” gene sets) enrichment analyses for DEGs in each cell type.

Supplementary Table S5 GO and KEGG enrichment analysis results for the four relative expression patterns.

Supplementary Table S6 Ion channel gene expression data extracted from the normalized AF dataset (GSE79768).

Supplementary Table S7 Differentially expressed ion channel genes and enrichment analysis results from the normalized AF dataset (GSE115574).

Supplementary Table S8 List of drugs significantly enriched in EF cell subgroups (e.g., phenytoin, medroxyprogesterone acetate) identified via gene concept network analysis.

Supplementary Figure S1 AF-specific cell-cell interaction signals (e.g., MHC-II, WNT, SEMA5).

Supplementary Figure S2 EF ligand-receptor pairs in AF vs. SR (e.g., PDGF, MDK up; LAMININ down).

Supplementary Figure S3 Weakened SMC→EC NPPA/B-NPR1 signaling in AF.

Supplementary Figure S4 SR-specific EC→APF signaling (GRN-SORT1, AGRN-DAG1).

Supplementary Figure S5 Signaling roles: EC/APF senders, EF/SMC receivers (key pair: MDK-NCL).

Supplementary Figure S6 SMC→EC NPPA-NPR1 network (dominant in SR).
