# Supplementary figures and images for: Cellular interactions and Ion channel signatures in atrial fibrillation remodeling: insights from single-cell analysis and machine learning
Source: Front Cardiovasc Med. 2025 Aug 15;12:1615574. doi: 10.3389/fcvm.2025.1615574 (PMC12394505; doi:10.3389/fcvm.2025.1615574)

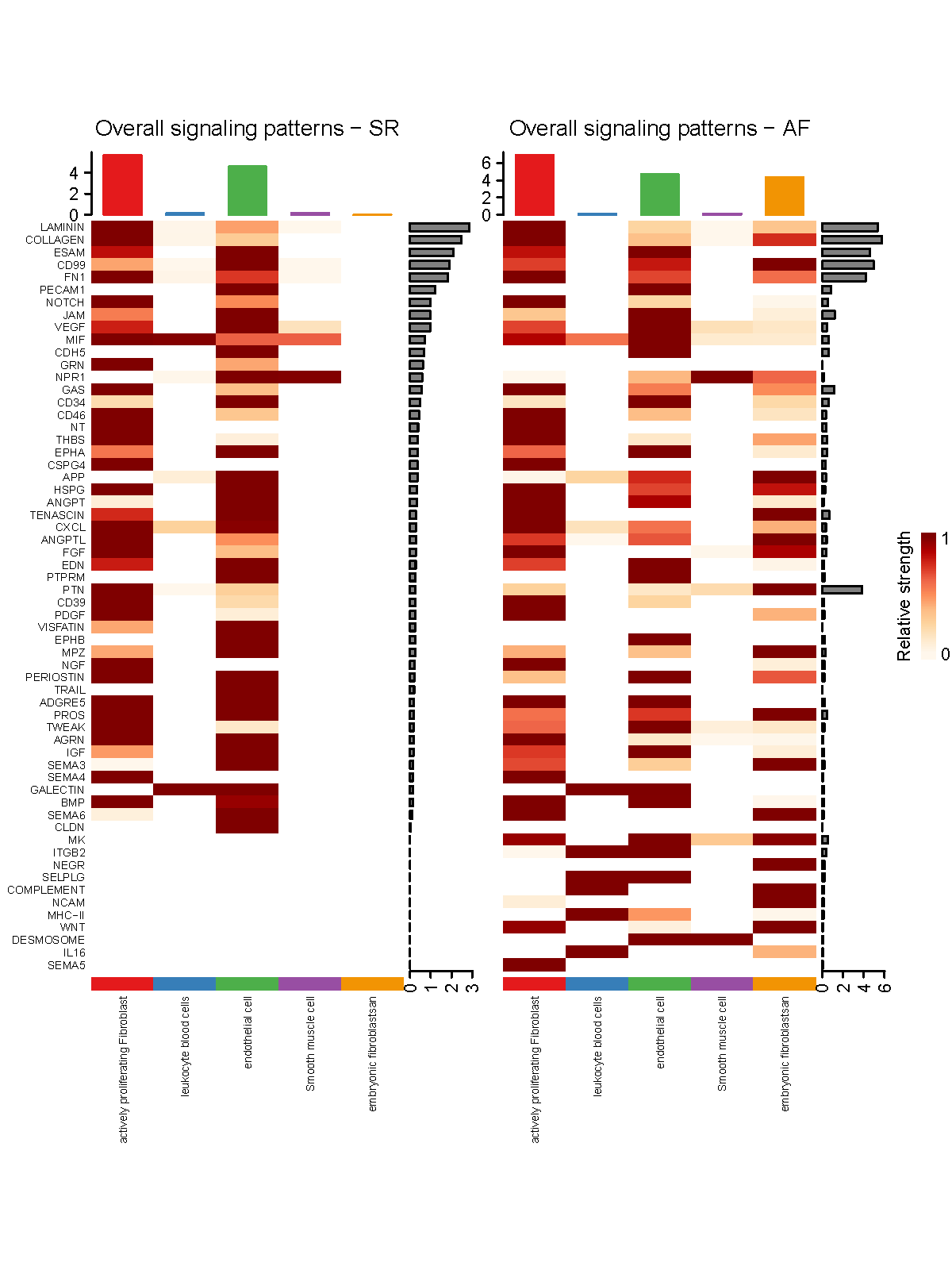

Supplement: Supplementary Figure S1 — AF-specific cell-cell interaction signals (e.g., MHC-II, WNT, SEMA5). [file Image1.tiff]

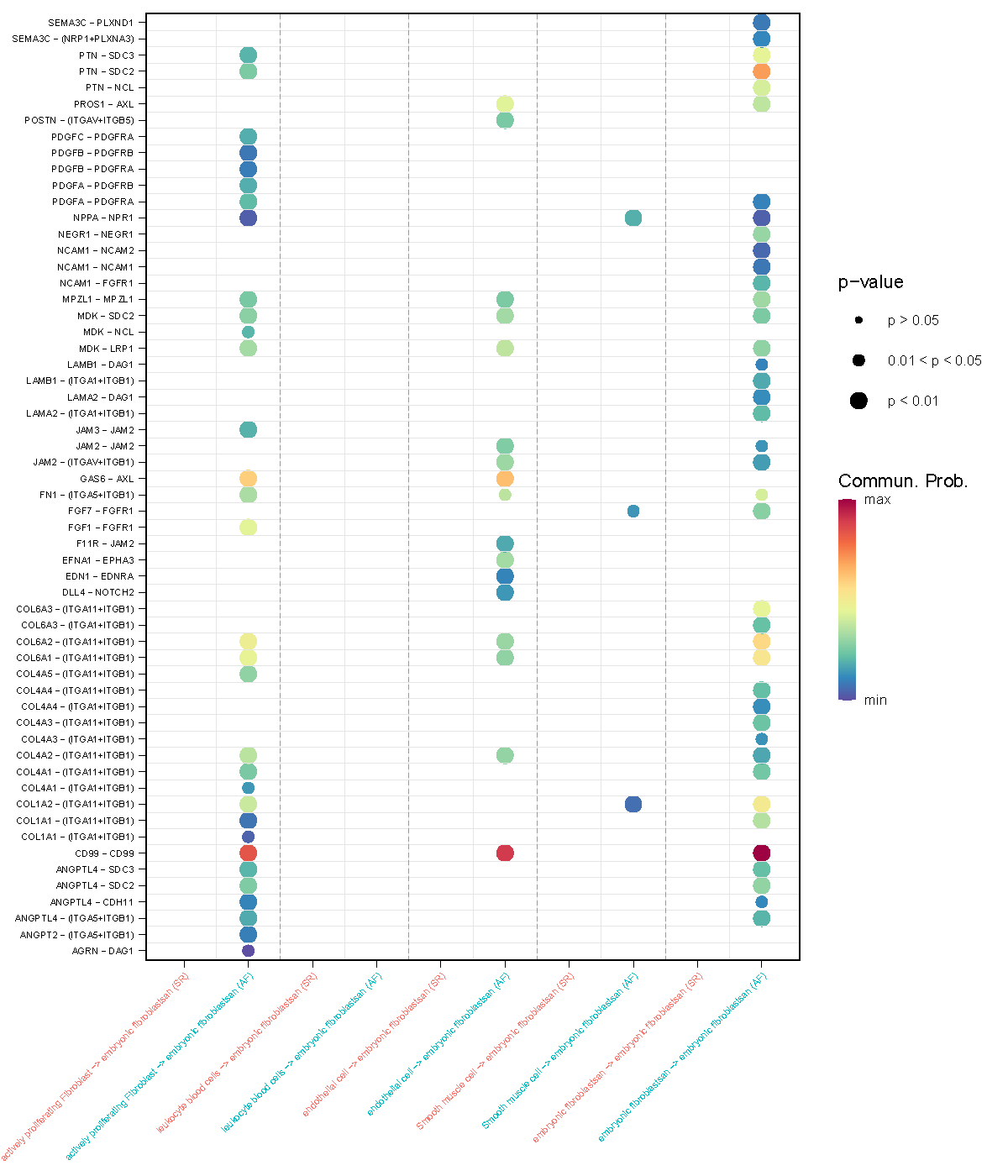

Supplement: Supplementary Figure S2 — EF ligand-receptor pairs in AF vs. SR (e.g., PDGF, MDK up; LAMININ down). [file Image2.tif]

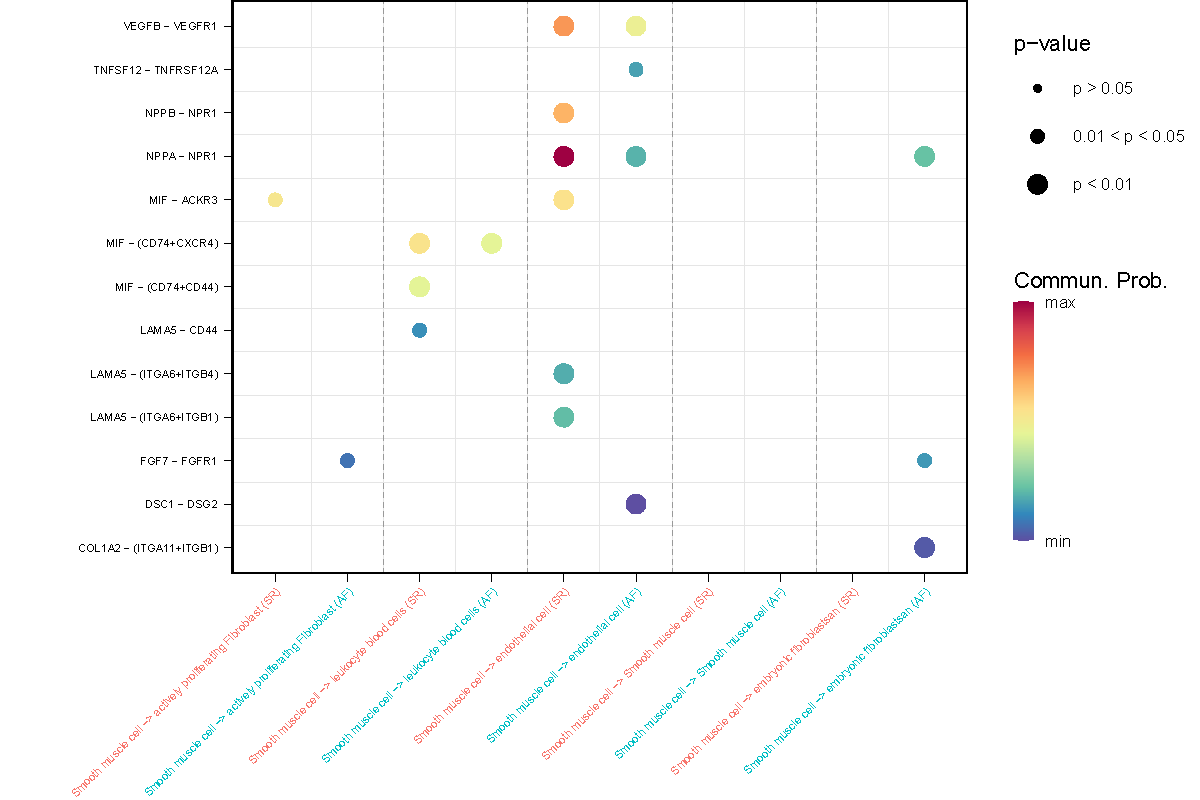

Supplement: Supplementary Figure S3 — Weakened SMC→EC NPPA/B-NPR1 signaling in AF. [file Image3.tif]

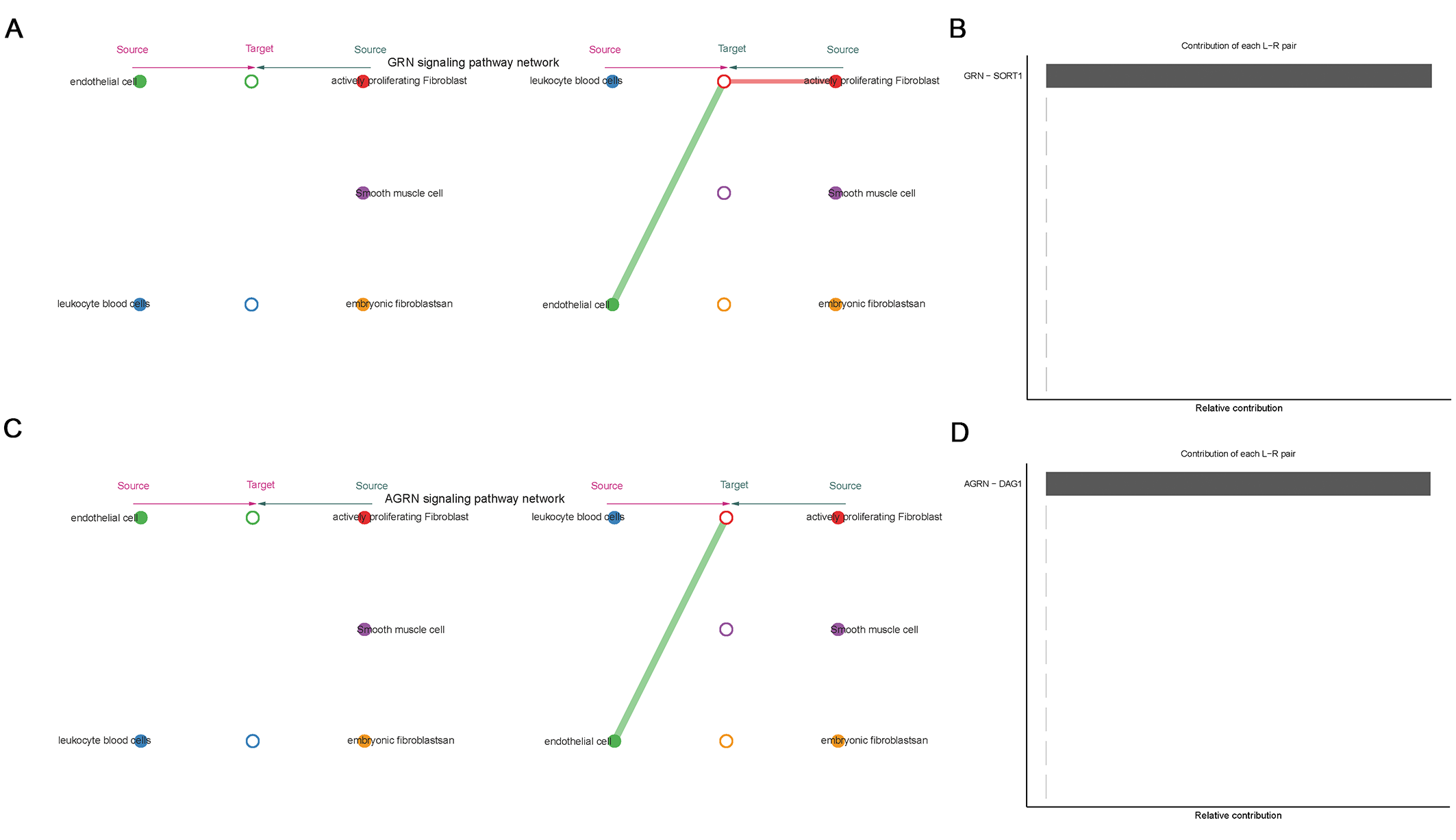

Supplement: Supplementary Figure S4 — SR-specific EC→APF signaling (GRN-SORT1, AGRN-DAG1). [file Image4.tif]

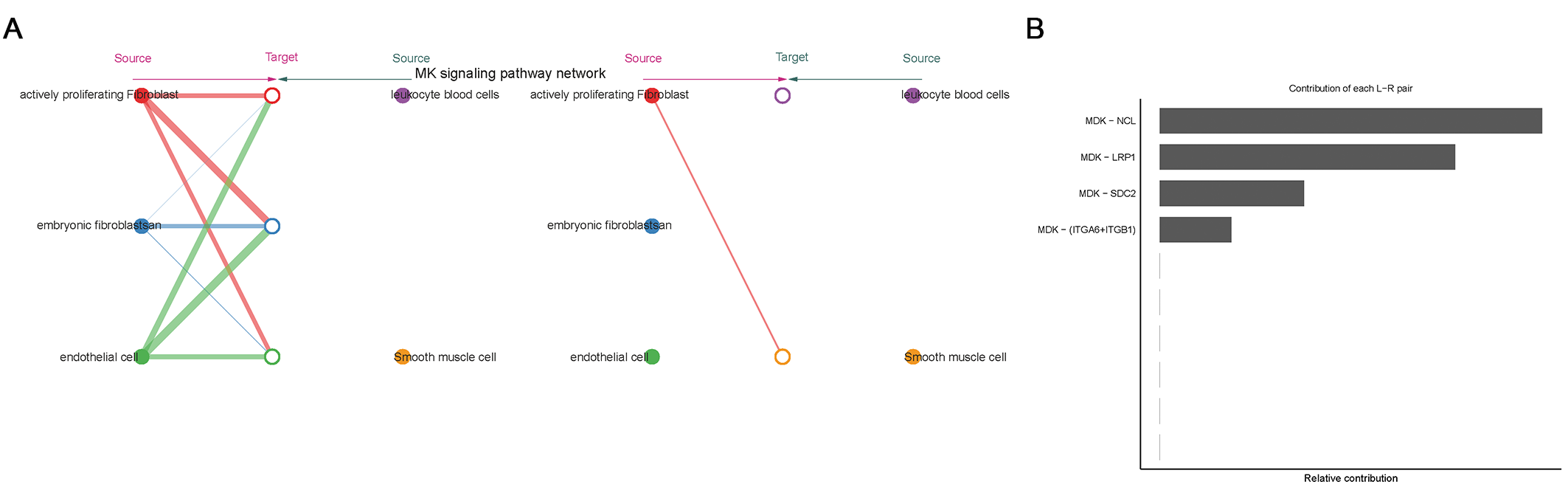

Supplement: Supplementary Figure S5 — Signaling roles: EC/APF senders, EF/SMC receivers (key pair: MDK-NCL). [file Image5.tif]

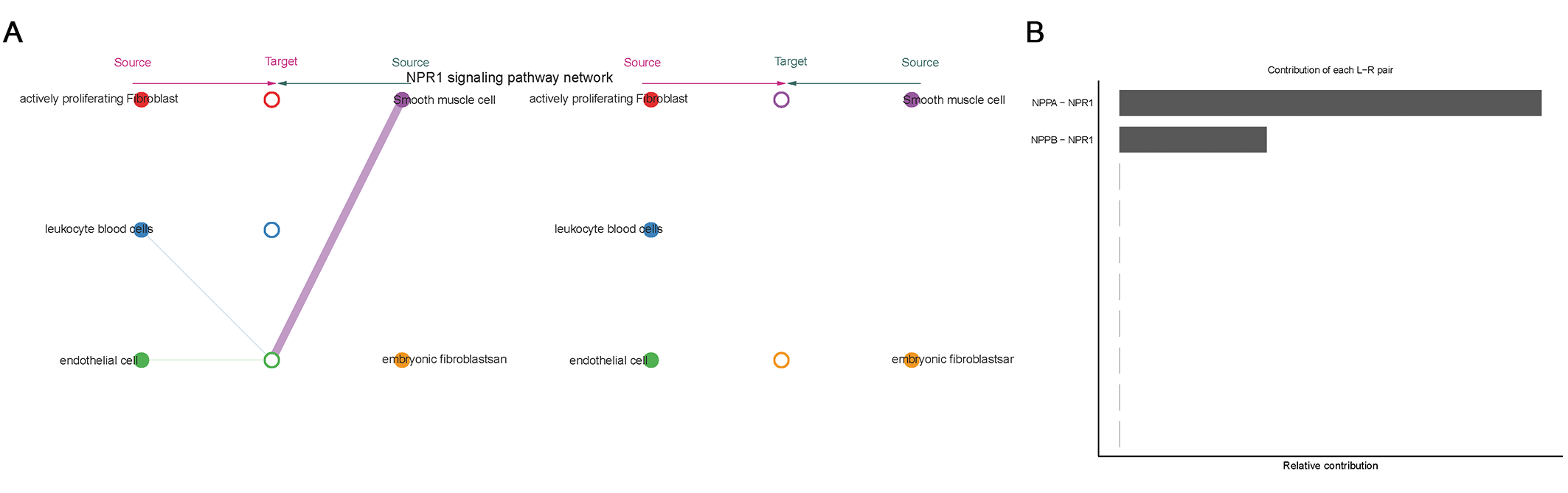

Supplement: Supplementary Figure S6 — SMC→EC NPPA-NPR1 network (dominant in SR). [file Image6.tif]
